# Recognition of unnatural base pairs by a eukaryotic DNA polymerase enables universal sequencing of an expanded genetic alphabet

**DOI:** 10.1093/nar/gkaf1460

**Published:** 2026-01-08

**Authors:** Hantao Luo, Yuhui Du, Leping Sun, Fangkai Ye, Jiezhao Ma, Xueting Wang, Yaxin Wang, Tingjian Chen

**Affiliations:** MOE International Joint Research Laboratory on Synthetic Biology and Medicines, School of Biology and Biological Engineering, South China University of Technology 510006, Guangzhou, China; MOE International Joint Research Laboratory on Synthetic Biology and Medicines, School of Biology and Biological Engineering, South China University of Technology 510006, Guangzhou, China; MOE International Joint Research Laboratory on Synthetic Biology and Medicines, School of Biology and Biological Engineering, South China University of Technology 510006, Guangzhou, China; MOE International Joint Research Laboratory on Synthetic Biology and Medicines, School of Biology and Biological Engineering, South China University of Technology 510006, Guangzhou, China; MOE International Joint Research Laboratory on Synthetic Biology and Medicines, School of Biology and Biological Engineering, South China University of Technology 510006, Guangzhou, China; MOE International Joint Research Laboratory on Synthetic Biology and Medicines, School of Biology and Biological Engineering, South China University of Technology 510006, Guangzhou, China; MOE International Joint Research Laboratory on Synthetic Biology and Medicines, School of Biology and Biological Engineering, South China University of Technology 510006, Guangzhou, China; MOE International Joint Research Laboratory on Synthetic Biology and Medicines, School of Biology and Biological Engineering, South China University of Technology 510006, Guangzhou, China

## Abstract

The expansion of the genetic alphabet through the development of unnatural base pairs (UBPs) has the potential to revolutionize synthetic biology and biotechnology. However, the replication of the UBPs by eukaryotic DNA polymerases is largely unexplored and the sequencing of the UBPs remains challenging. Herein, we explored and demonstrated the activity of human DNA polymerase *β* (Pol *β*) for the efficient and specific synthesis and extension of a panel of representative UBPs, including dNaM–dTPT3, dCNMO–dTPT3, and their functionalized derivatives. Based on this, we established a method for the sequencing of DNAs containing different unnatural bases, involving stalled primer extension mediated by Pol *β*, selective conversion of an unnatural nucleotide into two different natural ones in parallel and further primer extension mediated by Taq DNA polymerase, and Sanger or deep sequencing of the produced natural DNAs to locate the unnatural bases. The precision, universality, and potential for high-throughput applications of this method were demonstrated by the successful sequencing of various DNAs containing one or multiple of different unnatural bases. This work suggests the possibility of integrating the UBPs into the eukaryotic DNA replication systems and provides a technical foundation for the robust sequencing of DNAs with an expanded genetic alphabet.

## Graphical Abstract

**Graphical Abstract:**
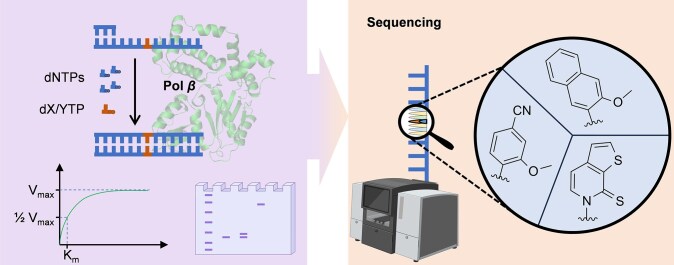

## Introduction

The natural genetic alphabet is composed of four nucleobases (A, T/U, G, and C), with which the fundamental script of life is written for literally all known living organisms. However, the limited number of natural bases greatly restricts their possible combinations, limiting the sequence and function diversity of the genetic polymers of life (i.e. DNA and RNA) [1]. Recent advances in the interface of chemistry and synthetic biology have led to the development of a series of unnatural base pairs (UBPs) that can be used for expanding the genetic alphabet [2]. Several representative UBPs, such as dP–dZ, dB–dS, dDs–dPx, and dNaM–dTPT3, have been reported to be efficiently replicated, amplified and transcribed by DNA and RNA polymerases *in vitro* [3–9]. dNaM–dTPT3 relies on hydrophobic and packing forces, and demonstrated significantly enhanced replication fidelity in polymerase chain reaction (PCR) amplification with OneTaq or Taq DNA polymerase, compared with earlier UBPs of the same type developed by Romesberg group, such as dNaM–d5SICS [7]. Moreover, dNaM–dTPT3, dCNMO–dTPT3, and their analogues have been successfully introduced into living cells of *Escherichia coli*, with their *in vivo* replication, transcription, and translation well achieved [10–12]. These achievements not only lead to a significant increase in the density of information stored in DNA but also provide revolutionary tools for the production of nucleic acids and proteins with increased building blocks, improved properties, and expanded functions, promoting the development of biotechnology, biomedicine, and biomaterials [2, 13–15].

Despite the great potential of the UBPs, there remain significant challenges to apply them to expand the genetic alphabet in eukaryotic cells. A few progresses have been made toward this goal in recent years. For example, Romesberg and coworkers successfully carried out the translation of NaM or TPT3-containing codons in eukaryotic cells, demonstrating the greater tolerance of the eukaryotic ribosome for the UBP compared to the prokaryotic ribosome [16]. Furthermore, Wang and coworkers demonstrated that eukaryotic RNA polymerase II can selectively recognize the UBPs and transcribe the dTPT3 nucleotide in a DNA template with rNaMTP with high fidelity, and provided important insights into the transcription process of these UBPs via structural analysis and molecular dynamics simulation [17]. Other than transcription and translation, the replication of the UBPs by eukaryotic DNA polymerases is also critical for their use in eukaryotes. Multiple distinct types of DNA polymerases have been identified in eukaryotic organisms, exhibiting significant functional diversity. For instance, DNA polymerases *α, β, ε*, and *δ* serve as essential components that play different roles for the replication and repair processes of the genomic DNA of eukaryotic cells [18, 19]. Previously, rat DNA polymerase *β* (Pol *β*) has proven to efficiently replicate DNA containing hydrophobic UBP d7AI–d7AI when combined with the Klenow fragment (KF) of *E. coli* DNA polymerase I, and human Pol *β* has been shown to be able to replicate hydrophobic UBP d5SICS–dMMO2 by itself [20, 21]. However, the investigation of the ability of eukaryotic DNA polymerases to recognize dNaM–dTPT3 and dCNMO–dTPT3, two representative UBPs that have been employed to construct the prokaryotic semi-synthetic organisms with an expanded genetic alphabet, is limited.

The development of sequencing technologies for DNAs with unnatural bases is essential for realizing the full potential of the UBPs. Traditionally, sequencing of DNAs containing unnatural bases relied on the termination of the fluorescent signal at the position of the unnatural base in the absence of the corresponding unnatural nucleoside triphosphate during Sanger sequencing [22]. However, this method was limited to the detection of a single unnatural base in the DNA sequence and could not be applied for efficient sequencing of DNA sequences containing multiple unnatural bases. The development of nanopore sequencing technology offered a powerful method that enabled the direct detection of unnatural bases with the MspA nanopore, albeit with limitations such as the complicated procedures and requirement of expensive apparatus [23]. Later, Li and coworkers developed a bridge-base approach, in which the dNaM–dTPT3 pair was transformed into a dG–dC pair with the assistance of bridge base isoTAT or into a dT–dA pair without the assistance of isoTAT, enabling the sequencing of DNAs containing multiple dNaM–dTPT3 pairs [24]. However, the requirement for a bridge base specifically designed for a certain UBP presented a potential constraint on its widespread application. Hirao group also successfully developed several methods for sequencing DNAs containing their UBPs. For example, a Sanger gap sequencing method was developed for sequencing DNAs containing the dDs–dPx pair by using modified dPx substrates to generate clear gaps at the positions of the dDs–dPx pairs in the sequencing spectrum [25]. In another method, they achieved sequencing of the dDs-containing DNAs via replacement PCR, in which the dDs bases were replaced with natural bases of different ratios due to different PCR processes [26]. Very recently, an inspiring approach has been reported by Benner group, which involved transliteration of dC to dU with a cytidine deaminase and subsequent transliteration of dZ–dP pair to dC–dG pair via the synthesis of unnatural–natural mispair during PCR, eliminating the need of a bridge base or modified unnatural triphosphate [27]. Nevertheless, this strategy required the transliteration of dZ to dC, which may not support its application in sequencing other types of UBPs.

In this study, we first explored the activity of human Pol *β* for the synthesis and extension of UBPs dNaM–dTPT3, dCNMO–dTPT3, and their functional derivatives. Then, we carried out steady-state kinetic experiments to rigorously characterize the efficiency and specificity of Pol *β* for the synthesis of these UBPs. The capability of Pol *β* for gap filling via the synthesis of one of the UBPs and subsequent strand-displacement primer extension was also investigated. Taking advantage of the high specificity of Pol *β* for the UBP synthesis, we established a method for sequencing DNAs containing unnatural bases, which involves the stalled primer extension mediated by Pol *β* and Taq DNA polymerase-mediated selective conversion of an unnatural nucleotide into two different natural nucleotides in parallel and further primer extension. The universality of this method was then demonstrated by sequencing various DNA oligonucleotides containing different unnatural bases.

## Materials and methods

### Materials

Plasmid pET30a(+)-Pol *β*, cyanine 3 (Cy3)-labeled DNA primer Cy3–T17, carboxyfluorescein (FAM)-labeled DNA primer FAM–T17, DNA oligonucleotides (Supplementary Table S1), bovine serum albumin (BSA), Ni-NTA sefinose resin 6FF (His-Tag), dATP, dTTP, dCTP, dGTP, ATP, and 2× TBE–urea sample buffer were purchased from Sangon Biotech Co., Ltd. (Shanghai, China). Lambda exonuclease, 10× standard Taq reaction buffer, 10× ThermoPol reaction buffer, T4 polynucleotide kinase (T4 PNK), and 10× T4 PNK reaction buffer were purchased from New England Biolabs (Ipswich, MA, USA). Unnatural nucleoside triphosphates 2-methoxy-3-(2-deoxy-β-d-erythro-pentofuranosyl)-naphthalene triphosphate (dNaMTP), 3-methoxy-4-(2-deoxy-β-d-erythro-pentofuranosyl)-benzonitrile triphosphate (dCNMOTP), (2-deoxy-β-d-erythro-pentofuranosyl)-thieno[3,4]pyridine-2-thione triphosphate (dTPT3TP), amino-modified dTPT3TP (dTPT3^Am^TP), and alkynyl-modified dTPT3TP (dTPT3^Al^TP) were purchased from WuXi AppTec (Wuxi, China). SFM4-3 (a mutant of the Stoffel fragment of Taq DNA polymerase) was prepared in the lab. Taq DNA polymerase was purchased from Coolaber Science Co., Ltd. (Beijing, China). Amicon Ultra centrifugal filters (MWCO 30 kDa and MWCO 50 kDa) were purchased from Sigma–Aldrich (St. Louis, MO, USA). Mixture of natural deoxyribonucleoside triphosphates (dNTPs), PrimeSTAR^®^ HS DNA polymerase, and 2× PrimeSTAR^®^ GC buffer were purchased from Takara Bio (Beijing, China). Zymo ssDNA/RNA Clean and Concentrator™ kit was purchased from Zymo Research (Irvine, CA, USA). Magen HiPure gel pure DNA micro kit was purchased from Magen Biotechnology (Guangzhou, China). Kanamycin was purchased from Solarbio Science and Technology Co., Ltd (Beijing, China). Cyber gold was purchased from Biolite Biotech Co., Ltd (Xi’an, China). 6× Loading buffer was purchased from Yeasen Biotechnology Co., Ltd (Shanghai, China).

### Expression and purification of Pol *β*

Expression plasmid pET30a(+)-Pol *β* was transformed into *E. coli* BL21(DE3) cells for the expression of Pol *β* (Supplementary Note S1). A single colony of *E. coli* BL21(DE3) cells harboring pET30a(+)-Pol *β* was inoculated into 2× YT medium supplemented with 50 μg/ml kanamycin and grown at 37°C overnight. The overnight culture was diluted 1:100 into fresh 2× YT medium supplemented with 50 μg/ml kanamycin and grown at 37°C. When the OD_600_ value of the culture reached 0.6, 0.25 mM isopropyl β-d-1-thiogalactopyranoside was added to induce the expression of Pol *β*, and then the culture was incubated at 37°C for 8 h. The cells were harvested by centrifugation at 3500 rpm and 4°C, and the pellet was resuspended in 1× binding buffer [50 mM Tris–HCl, 5 mM imidazole, 100 mM NaCl, 0.1 mM ethylenediaminetetraacetic acid (EDTA), pH 7.5] and disrupted with high-pressure homogenizer (ATS). The cell lysate was centrifuged at 10 000 rpm and 4°C for 60 min, and then the supernatant was filtered with 0.22 μm membrane filters. Pol *β* protein was purified successively by a Ni-NTA sefinose resin 6FF column and a DEAE sefinose column, concentrated to 0.3–0.5 mg/ml using an Amicon Ultra centrifugal filter (MWCO 30 kDa), and stored in a solution containing 20 mM Tris–HCl, 100 mM NaCl, 1 mM dithiothreitol (DTT), 1 mM EDTA, and 50% (v/v) glycerol (pH 7.5) at −20°C.

### Preparation of single-stranded DNA (ssDNA) oligonucleotides containing different unnatural bases

Oligonucleotides containing one or multiple dNaMs were purchased from Sangon Biotech Co., Ltd. (Shanghai, China), and those containing a dCNMO or dTPT3 were prepared by a one-pot enzymatic synthesis method as described previously with minor modifications [28]. Briefly, primer P32 was mixed with 5′-phosphorylated natural DNA template 5′P-T43 at a ratio of 1:2 in 1× standard Taq reaction buffer, incubated at 95°C for 10 min and slowly cooled down to room temperature to anneal the primer to the template. Then, 1 μM of the primer/template complex was mixed with 50 μM dCNMOTP or dTPT3TP, and 300 nM SFM4-3 in 1× ThermoPol reaction buffer, and the mixture was incubated at 37°C for 10 min or 3 h. After the incorporation of a dCNMO or dTPT3 nucleotide opposite the natural nucleotide in the template, 200 μM each of dNTPs was directly added into the reaction, followed by incubation at 37°C for 30 min. The products were purified with a Magen gel purification kit, and their concentrations were determined by a NanoDrop 2000 spectrophotometer. Then, 100 ng/μl products were incubated with 0.05 U lambda exonuclease in 1× lambda buffer at 37°C for 3 h to remove the 5′-phosphorylated natural DNA template. The oligonucleotide products were purified with a Zymo ssDNA/RNA Clean and Concentrator™ kit and stored at −20°C before used for later experiments.

### Incorporation of a single natural or unnatural nucleotide opposite a natural or unnatural nucleotide in the DNA template by Pol *β*

For the incorporation of a single natural or unnatural nucleotide opposite an unnatural nucleotide in the DNA template by Pol *β*, Cy3-labeled primer P17 was mixed with DNA template T55–NaM, T55–CNMO, or T55–TPT3 at a 1:2 ratio in 1× Pol *β* reaction buffer, incubated at 95°C for 10 min, and slowly cooled down to room temperature to anneal the primer to the template. Then, 20 nM of the primer/template complex was mixed with 10 or 100 μM dNaMTP, dCNMOTP, dTPT3TP, dTPT3^Am^TP, or dTPT3^Al^TP, or 10 or 100 μM each of dNTPs, 0.5 mg/ml BSA, 7% glycerol, and 100 nM Pol *β* in 1× Pol *β* reaction buffer and incubated at 37°C for 60 min. The reaction was quenched by mixing the reaction solution with an equal volume of 2× TBE–urea sample buffer, and incubated at 95°C for 10 min. All the products were analyzed with 20% denaturing polyacrylamide gel electrophoresis (PAGE) gels containing 8 M urea.

For the incorporation of a natural nucleotide opposite a natural nucleotide in the DNA template by Pol *β*, Cy3-labeled primer P17 was mixed with DNA template T55-A, T55-T, T55-C, or T55-G at a ratio of 1:2 in 1× Pol *β* reaction buffer, incubated at 95°C for 10 min, and slowly cooled down to room temperature to anneal the primer to the template. Then, 20 nM of the primer/template complex was mixed with 10 μM dATP, dTTP, dCTP, or dGTP, 0.5 mg/ml BSA, 7% glycerol, and 100 nM Pol *β* in 1× Pol *β* reaction buffer, and incubated at 37°C for 60 min. The reaction was quenched by mixing the reaction solution with an equal volume of 2× TBE–urea sample buffer and incubated at 95°C for 10 min. All the products were analyzed with 20% denaturing PAGE gels containing 8 M urea.

### Primer extension with natural and unnatural nucleoside triphosphates and a DNA template containing an unnatural base by Pol *β*

For the primer extension with dNTPs and with or without an unnatural nucleoside triphosphate by Pol *β*, 20 nM of the primer/template complex (P17/T55–NaM, P17/T55–CNMO, or P17/T55–TPT3) was mixed with 10 or 100 μM dNTPs, 10 or 100 μM dNaMTP, dCNMOTP, dTPT3TP, dTPT3^Am^TP, dTPT3^Al^TP, or none, 0.5 mg/ml BSA, 7% glycerol, and 100 nM Pol *β* in 1× Pol *β* reaction buffer, and incubated at 37°C for 60 min. The reaction was quenched by mixing the reaction solution with an equal volume of 2× TBE–urea sample buffer and incubated at 95°C for 10 min. All the products were analyzed with 20% denaturing PAGE gels containing 8 M urea.

### Mass spectrometry analysis of products of primer extension with natural and unnatural nucleoside triphosphates and a DNA template containing an unnatural base by Pol *β*

For the investigation of Pol *β*-mediated UBP synthesis under normal conditions for DNA replication, primer extension experiments were carried out by mixing 20 nM of the primer/template complex (P17/T55–NaM or P17/T55–TPT3) with 10 or 100 μM dNTPs, 10 or 100 μM dNaMTP or dCNMOTP, 10 or 100 μM dTPT3TP, 0.5 mg/ml BSA, 7% glycerol, and 100 nM Pol *β* in 1× Pol *β* reaction buffer, and incubated at 37°C for 60 min. The reaction was quenched by mixing the reaction solution with an equal volume of 2× TBE–urea sample buffer and incubated at 95°C for 10 min. All the products were sent to Sangon Biotech Co., Ltd. (Shanghai, China) for mass spectrometry analysis.

### Activity assay of Pol *β* for gap filling by the incorporation of different natural and unnatural nucleoside triphosphates opposite an unnatural nucleotide in the DNA template and subsequent strand-displacement primer extension

To prepare the double-stranded DNA (dsDNA) substrate with a 1-nt gap for the gap filling and strand-displacement primer extension by Pol *β*, single-stranded DNA (ssDNA) oligonucleotide P20 was first 5′-phosphorylated by mixing 6 μM P20, 1 mM ATP, and 0.2 U/μl T4 PNK in 1× T4 PNK buffer, and incubating the mixture at 37°C for 30 min. The produced 5′-phosphorylated ssDNA oligonucleotide, 5′-P-P20, was purified using a Zymo ssDNA/RNA Clean and Concentrator™ kit. Then, 1 μM 5′-FAM-labeled primer FAM-P17, 4 μM 5′-P-P20, and 2 μM template (T55–NaM, T55–CNMO, or T55–TPT3) were mixed in 1× Pol *β* reaction buffer, incubated at 95°C for 10 min, and slowly cooled down to room temperature to anneal FAM-P17 and 5′-P-P20 onto the template. For the gap-filling reaction, 20 nM of the prepared dsDNA substrate with a 1-nt gap was mixed with 10 μM dNaMTP, dCNMOTP, dTPT3TP, dTPT3^Am^TP, dTPT3^Al^TP, or 10 μM each of dNTPs, 5 mg/ml BSA, 7% glycerol, and 100 nM Pol *β* in 1× Pol *β* reaction buffer, and incubated at 37°C for 30 min. For the strand-displacement primer extension reaction, 20 nM of the prepared dsDNA substrate with a 1-nt gap was mixed with 10 μM each of dNTPs, 10 μM dNaMTP, dCNMOTP, dTPT3TP, dTPT3^Am^TP, dTPT3^Al^TP, or none, 0.5 mg/ml BSA, 7% glycerol, and 100 nM Pol *β* in 1× Pol *β* reaction buffer, and incubated at 37°C for 30 min. The reactions were quenched by the addition of an equal volume of 2× TBE–urea sample buffer and incubated at 95°C for 10 min. All the products were analyzed with 20% denaturing PAGE gels containing 8 M urea.

### Steady-state kinetic experiments for Pol *β*-mediated incorporation of a natural or unnatural nucleoside triphosphate opposite a natural or unnatural nucleotide in the DNA template

5′-FAM-labeled primer FAM-P17 was mixed with DNA template T55–T, T55–NaM, T55–CNMO, or T55–TPT3 at a ratio of 1:2 in 1× Pol *β* reaction buffer, incubated at 95°C for 10 min, and slowly cooled down to room temperature to anneal the primer to the template. Then, 20 nM of the primer/template complex was mixed with 0.5 μM–2 mM dATP, dTPT3TP, dNaMTP, or dCNMOTP, and 0.5–5 nM Pol *β* in 1× Pol *β* reaction buffer and incubated at 37°C for different times. The reaction was quenched by the addition of an equal volume of 2× TBE–urea sample buffer and incubated at 95°C for 10 min. The products were analyzed with 20% denaturing PAGE gels containing 8 M urea. The gel bands were quantified by ImageJ software, and *K*_m_ and *k*_cat_ values were calculated by fitting the data to the Michaelis–Menten equation with GraphPad Prism 9 software. All experiments were triplicated.

### Stalled primer extension mediated by Pol *β*

For Pol *β*-mediated sequencing in which the original ssDNA oligonucleotide was initially phosphorylated at the 5′ end and removed by lambda exonuclease degradation after the selective conversion and further primer extension by Taq DNA polymerase, the template was first 5′-phosphorylated by mixing 1 μM of the template, 1 mM ATP, and 0.2 U/μl T4 PNK in 1× T4 PNK buffer and incubating the mixture at 37°C for 30 min. 5′-FAM-labeled primer FAM-P24-2 was mixed with DNA template T55–NaM, T55–CNMO, or T55–TPT3; 5′-FAM-labeled primer FAM-P24-1 was mixed with DNA template T71(TXA)-NaM, T71(CXG)-NaM, T79-NaM, or LIB-NaM; 5′-FAM-labeled primer FAM-P24-3 was mixed with DNA template T70(TXC)-NaM; 5′-FAM-labeled primer FAM-P24-4 was mixed with DNA template T70(AXT)-NaM; and 5′-FAM-labeled primer FAM-P24-5 was mixed with DNA template T40(GXC)-NaM (Supplementary Table S1) at a ratio of 2:1 in 1× Pol *β* reaction buffer. The mixtures were incubated at 95°C for 10 min and slowly cooled down to room temperature to anneal the primer to the template. Then, 0.1 μM of the primer/template complex was mixed with 10 μM dTPT3TP (only when T79–NaM was used as the template), 10 μM each of dNTPs, 0.5 mg/ml BSA, 7% glycerol, and 300 nM Pol *β* in 1× Pol *β* reaction buffer, and incubated at 37°C for 20 min. An aliquot of the product was saved for gel analysis, and the rest was purified with a Zymo ssDNA/RNA Clean and Concentrator™ kit and stored at −20°C before used for later experiments.

### Selective conversion of each of the unnatural nucleotide into a natural nucleotide, further primer extension, and PCR amplification of the primer extension product by Taq DNA polymerase

For the selective conversion of each of the unnatural nucleotide into a natural nucleotide, 0.4 ng/μl of the stalled primer extension product was mixed with 200 μM dATP (for the selective conversion of dNaM or dCNMO) or dTTP (for the selective conversion of dTPT3), or 200 μM dCTP, and 10 nM Taq DNA polymerase in 1× standard Taq reaction buffer, and incubated at 68°C for 20 min. For further primer extension, the selective conversion product was mixed with 200 μM each of the other three dNTPs and incubated at 68°C for 5 min. An aliquot of the product was saved for gel analysis, and the rest was mixed with 0.05 U/μl lamda exonuclease and incubated at 37°C for 30 min (for those from experiments in which the original ssDNA oligonucleotide was initially phosphorylated at the 5′ end) or left untreated (for the others). Then the product was mixed with 1 μM primer FAM-P24-1, FAM-P24-2, FAM-P24-3, FAM-P24-4, or FAM-P24-5, and 1 μM primer P27-1, P27-2, P27-3, P27-4, or P27-5 (Supplementary Table S1), and was amplified by PCR with the following thermocycling program: 94°C, 2 min, 35 cycles of (94°C, 20 s; 58°C, 20 s; 68°C, 45 s), 68°C, 5 min. The product was analyzed by a 6% PAGE gel, and the gel was stained with Cyber Gold. The target products were separated by cutting the gel and purified with a Magen gel purification kit.

### Sanger sequencing of the PCR products (natural dsDNA oligonucleotides) produced from the oligonucleotides containing one or multiple unnatural bases for their sequencing mediated by Pol *β*

For Sanger sequencing, dsDNA arms, which were used for lengthening the PCR products produced from oligonucleotides containing one or multiple unnatural bases as described in the previous section, were PCR amplified from plasmid pET30a(+). Each of the PCR mixture was prepared by mixing 1 μM primer P47-1, P47-2, P47-3, P47-4, or P47-5, 1 μM primer P20-30a (Supplementary Table S1), 1 ng/μl plasmid pET30a(+), 200 μM each of dNTPs, and 0.025 U/μl PrimeSTAR^®^ HS DNA polymerase in 1× PrimeSTAR^®^ GC buffer. The PCR reaction was carried out with the following thermocycling program: 94°C, 30 s, 32 cycles of (94°C, 20 s; 55°C, 20 s; 68°C, 90 s), 68°C, 5 min. Then the produced dsDNA arms were purified with a Zymo ssDNA/RNA Clean and Concentrator™ kit. To attach a dsDNA arm to one of the PCR products produced from oligonucleotides containing one or multiple unnatural bases, overlap PCR was carried out by mixing 10 ng/μl of the dsDNA arm, 2 ng/μl of the PCR product, 200 μM each of dNTPs, and 0.025 U/μl PrimeSTAR^®^ HS DNA polymerase in 1× PrimeSTAR^®^ GC buffer. The first stage of the overlap PCR was carried out with the following thermocycling program: 94°C, 30 s, 15 cycles of (94°C, 20 s; 60°C, 30 s; 68°C, 1 min), 68°C, 5 min. After the addition of 1 μM primer FAM-P24-1, FAM-P24-2, FAM-P24-3, FAM-P24-4, or FAM-P24-5 and 1 μM primer P20-30a into the overlap PCR mixture, the second stage of the overlap PCR was carried out with the following thermocycling program: 94°C, 30s, 22 cycles of (94°C, 20 s; 60°C, 30 s; 68°C, 1 min), 68°C, 5 min. The overlap PCR products were sent to Sangon Biotech Co., Ltd. (Shanghai, China) for purification and Sanger sequencing. The calculation of the peak areas for different bases in the sequence spectra was carried out with R package sangerseqR.

### Deep sequencing of the PCR products (natural dsDNA oligonucleotides) produced from the DNA oligonucleotides containing one or multiple unnatural bases for their sequencing mediated by Pol *β*

To prepare each of the samples for deep sequencing, a PCR reaction solution was prepared by mixing 0.5 μM barcode-containing upstream primer [Deep-T55NaM-A, Deep-T55NaM-C, Deep-T55CNMO-A, Deep-T55CNMO-C, Deep-T55TPT3-T, Deep-T55TPT3-C, Deep-T40(GXC)-A, Deep-T40(GXC)-C, Deep-T71(CXG)-A, Deep-T71(CXG)-C, Deep-T70(TXC)-A, Deep-T70(TXC)-C, Deep-T70(AXT)-A, Deep-T70(AXT)-C, Deep-T71(TXA)-A, Deep-T71(TXA)-C, Deep-T79-A, Deep-T79-C, Deep-LIB-A, or Deep-LIB-C], 0.5 μM downstream primer [Deep-T55-Down, Deep-T40(GXC)-Down, Deep-T71(CXG)-Down, Deep-T70(TXC)-Down, Deep-T70(AXT)-Down, or Deep-P17] (Supplementary Table S1), 0.1 ng/μl of the overlap PCR product, 200 μM each of dNTPs, and 0.025 U/μl PrimeSTAR^®^ HS DNA polymerase in 1× PrimeSTAR^®^ GC buffer. The PCR reaction was carried out with the following thermocycling program: 94°C, 30 s, 35 cycles of (94°C, 20s; 60°C, 20 s; 68°C, 90 s), 68°C, 5 min. The PCR products were mixed, concentrated to a total concentration of 40 ng/μl, and sent for deep sequencing.

## Results and discussion

### Recognition of a panel of UBPs by Pol *β*

**Figure 1.**
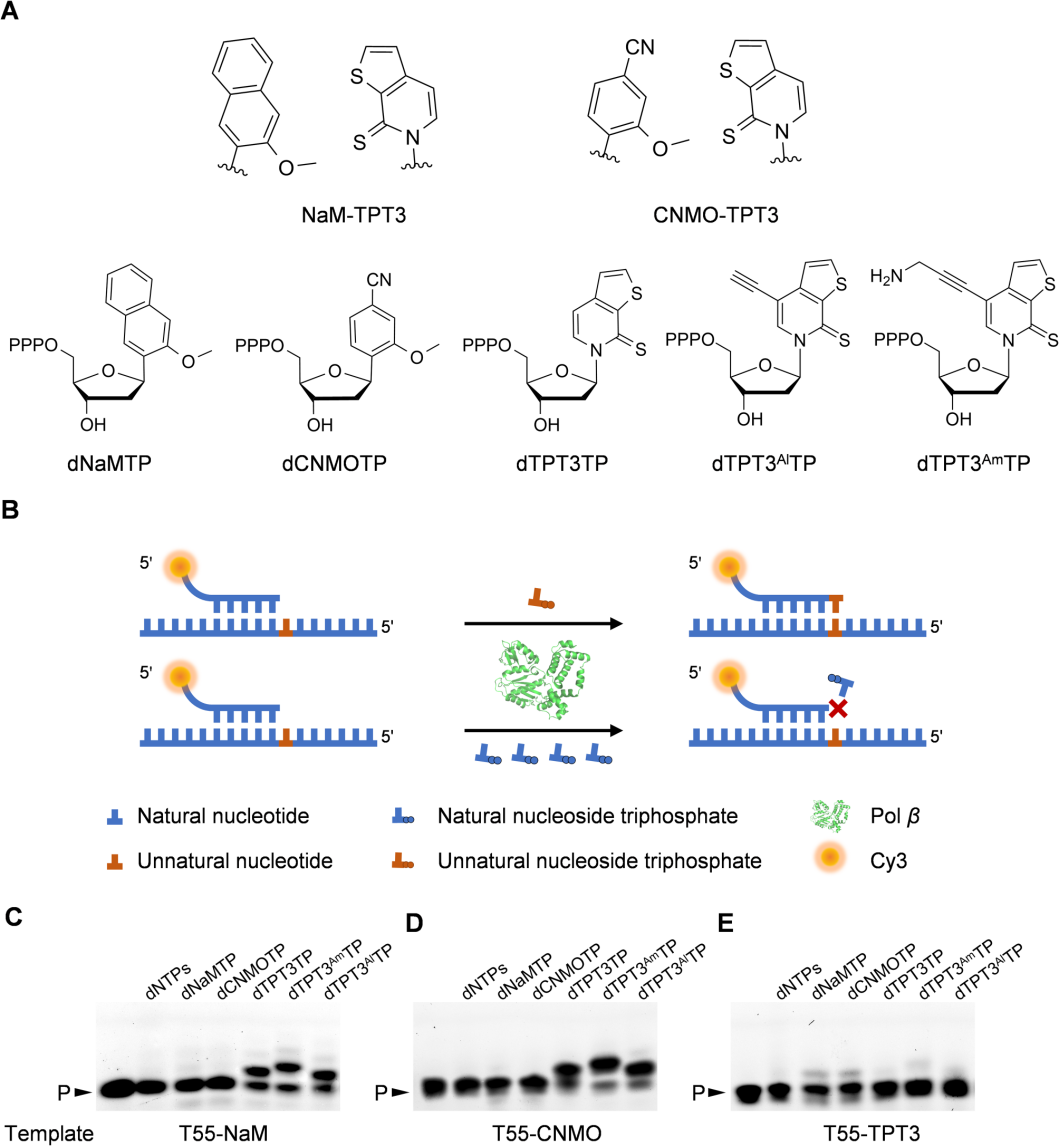
Pol *β*-mediated incorporation of a single natural or unnatural nucleotide opposite an unnatural nucleotide in the DNA template. (**A**) Chemical structures of UBPs NaM–TPT3 and CNMO–TPT3, and unnatural nucleoside triphosphates dNaMTP, dCNMOTP, dTPT3TP, dTPT3^Al^TP, and dTPT3^Am^TP involved in this study. (**B**) Scheme of the single-nucleotide incorporation experiments. (**C–E**) Single-nucleotide incorporation using a DNA template containing a dNaM, dCNMO, or dTPT3 nucleotide (T55–NaM, T55–CNMO, or T55–TPT3). For each reaction, 20 nM primer/template complex was mixed with 10 μM dNaMTP, dCNMOTP, dTPT3TP, dTPT3^Am^TP, or dTPT3^Al^TP, or 10 μM each of dNTPs, 0.5 mg/ml BSA, 7% glycerol, and 100 nM Pol *β* in 1× Pol *β* reaction buffer and incubated at 37°C for 60 min. The products were analyzed with 20% denaturing PAGE gels supplemented with 8 M urea. P: primer.

A panel of UBPs based on hydrophobic and packing forces, typified by dNaM–dTPT3 and dCNMO–dTPT3 (Fig. 1A), has been reported to be able to be replicated by various DNA polymerases from prokaryotic organisms and can be used for effective storage and retrieval of increased genetic information in *E. coli* [7, 12, 29, 30]. However, the activity of eukaryotic DNA polymerases to replicate these UBPs is less studied. In this study, we first explored the activity of Pol *β*, a single-subunit eukaryotic DNA polymerase that mainly participates in the DNA base-excision repair (BER) process [31, 32], to synthesize different UBPs through a single-nucleotide incorporation assay (Fig. 1B). A Cy3-labeled primer, Cy3-P17 was annealed to DNA template T55–NaM, T55–CNMO, or T55–TPT3 (Supplementary Table S1), which contained a dNaM, dCNMO, or dTPT3 nucleotide at the 5′ position adjacent to the nucleotide opposite the 3′ nucleotide of the primer. Then the primer was extended by mixing the primer/template complex with 10 μM each of dNTPs or 10 μM of one of the unnatural nucleoside triphosphates and 100 nM Pol *β* in 1× Pol *β* reaction buffer and incubating at 37°C for 60 min. As shown in Fig. 1C and D, when T55–NaM or T55–CNMO was used as the template, only dTPT3TP, dTPT3^Am^TP, and dTPT3^Al^TP were efficiently incorporated opposite the dNaM or dCNMO in the template, yielding extended primers incorporating one 3′ nucleotide with comparable efficiencies. No production of dNaM–dNaM or dCNMO–dCNMO self-pair was detected, nor was the production of any mispairs between the natural bases and dNaM or dCNMO, indicating the good specificity of Pol *β*-mediated synthesis of dNaM–dTPT3, dCNMO–dTPT3, and their functionalized derivatives. Remarkably, under the same experimental conditions, dTPT3TP and its derivatives were incorporated more efficiently opposite dCNMO than opposite dNaM, suggesting dCNMO–dTPT3 and its derivatives are better recognized by Pol *β* than dNaM–dTPT3 and its derivatives. When T55–TPT3 was used as the template, the production of dTPT3–dNaM and dTPT3–dCNMO was observed, albeit with lower efficiency than the production of dNaM–dTPT3 and dCNMO–dTPT3 using template T55–NaM and T55–CNMO, respectively (Fig. 1E). Unexpected incorporation of dTPT3^Am^TP opposite the dTPT3 nucleotide was also observed, but less efficient than the incorporation of dNaMTP and dCNMOTP opposite the dTPT3 nucleotide. Comparison of the syntheses of the UBPs and natural base pairs revealed that the synthesis efficiencies of dNaM–dTPT3 and its analogues were not much less than those of the natural base pairs, and the synthesis efficiencies of dCNMO–dTPT3 and its analogues were almost identical to those of the natural base pairs under the same reaction conditions (Fig. 1C and D, and Supplementary Fig. S1). We also carried out single-nucleotide incorporation experiment with an increased concentration of each of the dNTPs or the unnatural nucleoside triphosphate (100 μM). As shown in Supplementary Fig. S2, under this condition, the synthesis efficiencies of dNaM–dTPT3/dTPT3^Am^/dTPT3^Al^, dCNMO–dTPT3/dTPT3^Am^/dTPT3^Al^, dTPT3–dNaM, and dTPT3–dCNMO were significantly increased. Although the synthesis efficiencies of the mispairs were also increased, they were still significantly lower than those of the correct UBPs. These results together suggest that Pol *β* is capable of synthesizing dNaM–dTPT3, dCNMO–dTPT3, and their functionalized derivatives with good efficiency and specificity and synthesizing dTPT3–dNaM and dTPT3–dCNMO with lower efficiency and specificity.

**Figure 2.**
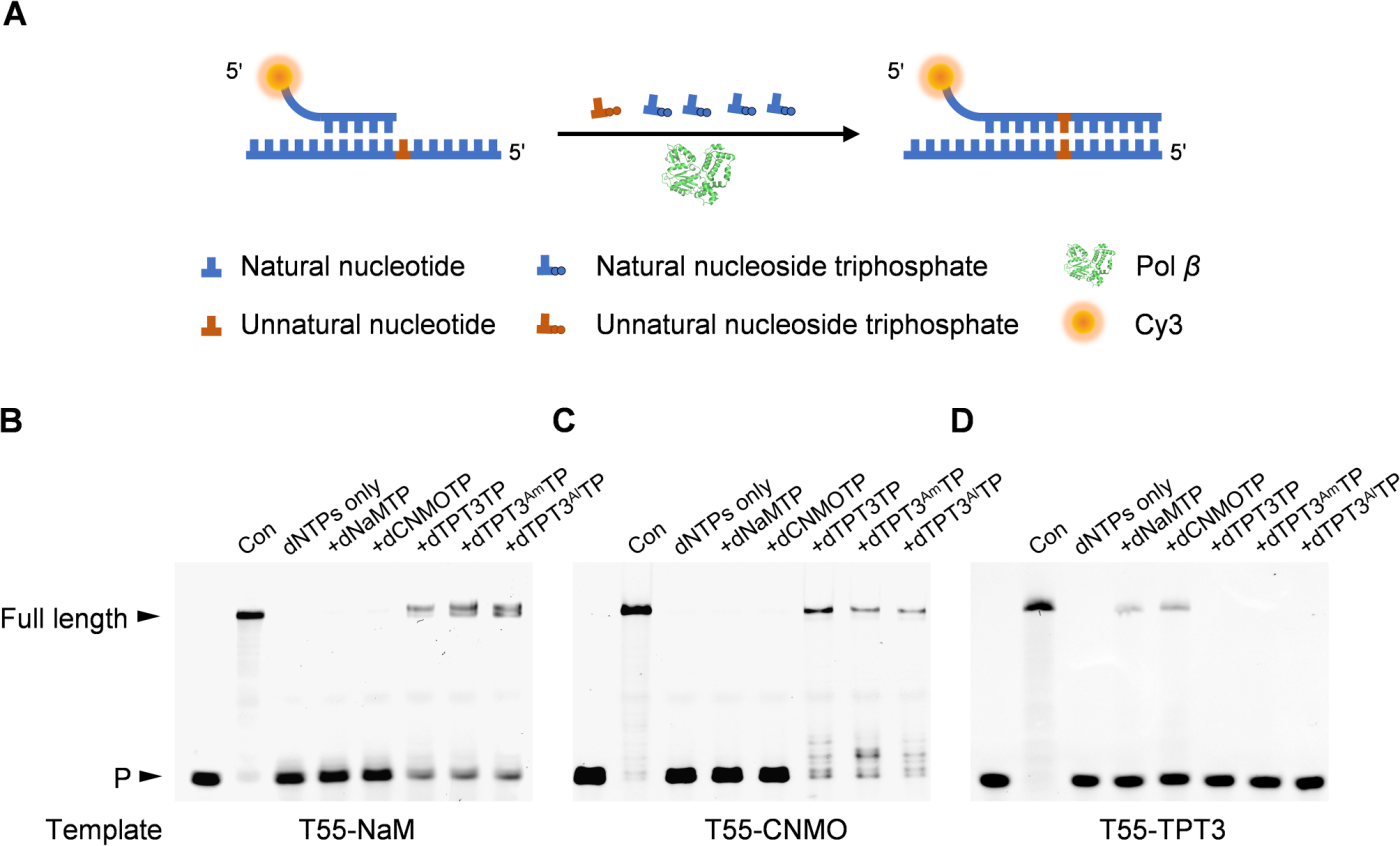
Pol *β*-mediated primer extension with unnatural nucleotide-containing DNA templates and natural and unnatural nucleoside triphosphates. (**A**) Scheme of the primer extension experiments. (**B–D**) Pol *β*-mediated primer extension with DNA templates containing a dNaM, dCNMO, or dTPT3 nucleotide (T55–NaM, T55–CNMO or T55–TPT3) and natural and unnatural nucleoside triphosphates. For each reaction, 20 nM primer/template complex was mixed with 10 μM each of dNTPs, 10 μM dNaMTP, dCNMOTP, dTPT3TP, dTPT3^Am^TP, dTPT3^Al^TP, or none, 0.5 mg/ml BSA, 7% glycerol, and 100 nM Pol *β* in 1× Pol *β* reaction buffer and incubated at 37°C for 60 min. The products were analyzed with 20% denaturing PAGE gels supplemented with 8 M urea. Con: primer extension with a natural DNA template and dNTPs by Pol *β* under the same reaction conditions (control). P: primer.

Next, we explored the activity of Pol *β* in further extending the primer to the full length after the incorporation of an unnatural nucleotide opposite the unnatural nucleotide in the template (Fig. 2A). Primer Cy3-P17 was annealed to DNA template T55–NaM, T55–CNMO, or T55–TPT3 (Supplementary Table S1) and extended by mixing the primer/template complex with 10 μM each of dNTPs, 10 μM of one of the unnatural nucleoside triphosphates or none, and 100 nM Pol *β* in 1× Pol *β* reaction buffer and incubating at 37°C for 60 min. As shown in Fig. 2B and C, when T55–NaM or T55–CNMO was used as the template, Pol *β* was able to extend the primer to the full length in the presence of dNTPs and dTPT3TP, dTPT3^Am^TP, or dTPT3^Al^TP. In sharp contrast, in the absence of dTPT3TP, dTPT3^Am^TP, and dTPT3^Al^TP, no matter whether dNaMTP or dCNMOTP was added, no obvious primer extension was observed. This suggests that Pol *β* is able to extend dNaM–dTPT3, dCNMO–dTPT3, and their functionalized derivatives after synthesizing these UBPs with good specificity. It is worth noting that when template T55–CNMO was used, a larger proportion of the primer was extended compared to when template T55–NaM used, again suggesting that dCNMO–dTPT3 and its derivatives are more readily recognized by Pol *β*. When T55–TPT3 was used as the template, in the presence of dNTPs and dNaMTP or dCNMOTP, some full-length primer extension product was observed, while no obvious full-length primer extension product was observed in the absence of dNaMTP and dCNMOTP, no matter whether dTPT3TP, dTPT3^Am^TP, or dTPT3^Al^TP was added (Fig. 2D). This suggests the good specificity of Pol *β*-mediated synthesis and extension of dTPT3–dNaM and dTPT3–dCNMO. When the concentrations of each of the dNTPs and the unnatural nucleoside triphosphate were increased to 100 μM, primer extensions with all three templates yielded greater quantities of the full-length products after the synthesis of the correctly pairing UBPs (Supplementary Fig. S3). Remarkably, when template T55–CNMO was used, almost all the primer was extended after the synthesis of dCNMO–dTPT3, dCNMO–dTPT3^Am^, and dCNMO–dTPT3^Al^, further suggesting the good recognition of these UBPs by Pol *β*. When T55–TPT3 was used as the template, although more full-length primer extension product was produced in the presence of dNTPs and dNaMTP or dCNMOTP compared to in the absence of dNaMTP and dCNMOTP, in the presence of dTPT3TP, dTPT3^Am^TP, or dTPT3^Al^TP, a small amount of full-length primer extension product was also observed, presumably due to the nonspecific synthesis and extension of dTPT3–dTPT3 self-pair and its derivatives. This suggests that the specificity for Pol *β*-mediated synthesis and extension of dNaM–dTPT3 and dCNMO–dTPT3 is higher than that for Pol *β*-mediated synthesis and extension of dTPT3–dNaM and dTPT3–dCNMO. To investigate whether Pol *β* is able to efficiently and specifically synthesize the UBPs under normal conditions for DNA replication and to evaluate the possible generation of an unnatural self-pair (e.g. dNaM–dNaM and dTPT3–dTPT3) under these conditions, we carried out primer extension experiments with a dNaM-containing template (T55–NaM), dNTPs, dNaMTP, and dTPT3TP, or with a dTPT3-containing template (T55–TPT3), dNTPs, dNaMTP/dCNMOTP, and dTPT3TP and analyzed the products by mass spectrometry (Supplementary Fig. S4). As shown in Supplementary Fig. S4A, for each of the primer extension reactions with template T55–NaM, all of the product contained the correctly incorporated dTPT3, and no product containing a mismatched base or losing a base was observed. For each of the primer extension reactions with template T55–TPT3, although a substantial amount of the product contained the correctly incorporated dNaM or dCNMO, 25.70%–75.47% of the total product was found to have no unnatural nucleotide incorporated under the different tested conditions (Supplementary Fig. S4C–F), suggesting a need for efforts on improving the incorporation efficiencies of dNaMTP and dCNMOTP opposite a dTPT3 under these conditions in the future. In all cases, no detectable generation of an unnatural self-pair was observed. Overall, Pol *β* demonstrates good or moderate efficiency and specificity for the synthesis and extension of dNaM–dTPT3, dCNMO–dTPT3, functionalized derivatives of dNaM–dTPT3 and dCNMO–dTPT3, dTPT3–dNaM, and dTPT3–dCNMO, suggesting the possibility of integrating these UBPs into the eukaryotic DNA replication systems.

### Pol *β*-mediated gap filling by the incorporation of an unnatural nucleotide opposite an unnatural nucleotide in the template and subsequent strand-displacement primer extension

**Figure 3.**
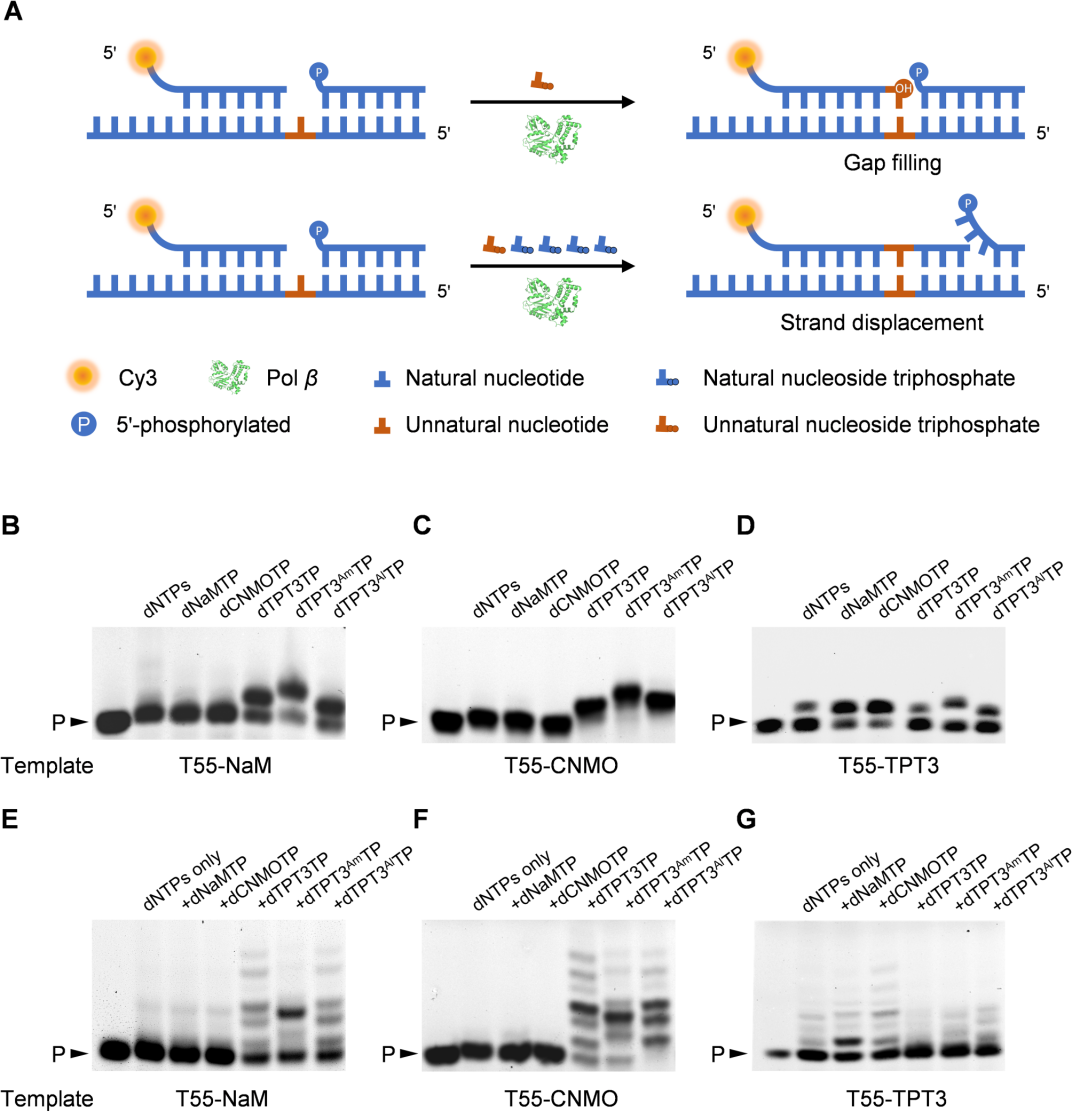
Pol *β*-mediated gap filling via the incorporation of a varying unnatural nucleoside triphosphate opposite an unnatural nucleotide in the DNA template and subsequent strand-displacement primer extension. (**A**) Scheme of the experiment. (**B–D**) Pol *β*-mediated gap filling via the incorporation of a varying unnatural nucleoside triphosphate opposite the dNaM, dCNMO, or dTPT3 nucleotide in the DNA template (T55–NaM, T55–CNMO or T55–TPT3). For each reaction, 20 nM dsDNA substrate with a 1-nt gap was mixed with 10 μM dNaMTP, dCNMOTP, dTPT3TP, dTPT3^Am^TP, or dTPT3^Al^TP, or 10 μM each of dNTPs, 0.5 mg/ml BSA, 7% glycerol, and 100 nM Pol *β* in 1× Pol *β* reaction buffer and incubated at 37°C for 30 min. The products were analyzed with 20% denaturing PAGE gels supplemented with 8 M urea. P: primer. (**E–G**) Pol *β*-mediated strand-displacement primer extension after the incorporation of a varying unnatural nucleoside triphosphate opposite the dNaM, dCNMO, or dTPT3 nucleotide in the DNA template (T55–NaM, T55–CNMO, or T55–TPT3). For each reaction, 20 nM dsDNA substrate with a 1-nt gap was mixed with 10 μM each of dNTPs, 10 μM dNaMTP, dCNMOTP, dTPT3TP, dTPT3^Am^TP, dTPT3^Al^TP, or none, 0.5 mg/ml BSA, 7% glycerol, and 100 nM Pol *β* in 1× Pol *β* reaction buffer and incubated at 37°C for 30 min. The products were analyzed with 20% denaturing PAGE gels supplemented with 8 M urea. P: primer.

Pol *β* has been found to play an essential role in gap filling and strand-displacement DNA synthesis in mammalian BER [31, 32]. With Pol *β*’s good activity for the synthesis and extension of UBPs demonstrated, we further investigated its activity for gap filling by the incorporation of an unnatural nucleotide opposite an unnatural nucleotide in the template and subsequent strand-displacement primer extension to test whether the UBPs are compatible with the eukaryotic DNA repair machinery. For the gap-filling experiment, Cy3-labeled primer P17 and 5′-phosphorylated ssDNA oligonucleotide P20 were co-annealed to template T55–NaM, T55–CNMO, or T55–TPT3 (Supplementary Table S1), producing the dsDNA substrate containing a 1-nt gap opposite the unnatural nucleotide in the template (Fig. 3A). Then the gap-filling reaction was carried out by mixing the dsDNA substrate with a 1-nt gap, 10 μM each of dNTPs or 10 μM of one of the unnatural nucleoside triphosphates, and 100 nM Pol *β* in 1× Pol *β* reaction buffer and incubating at 37°C for 30 min. As shown in Fig. 3B-D, Pol *β* efficiently performed the gap filling by specifically incorporating a dTPT3, dTPT3^Am^, or dTPT3^Al^ opposite the dNaM or dCNMO in the template, and a dNaM or dCNMO opposite the dTPT3 in the template. Incorporation of a natural nucleotide, dTPT3, dTPT3^Am^, or dTPT3^Al^ opposite the dTPT3 in the template was also observed, albeit with much less efficiency compared to the incorporation of a dNaM or dCNMO. This indicates that Pol *β* is also able to fill the gap via the synthesis of a dTPT3-natural mispair, a dTPT3–dTPT3 self-pair, or one of the analogues of the dTPT3–dTPT3 self-pair. In all cases, for the extended primer, no more than one nucleotide was incorporated, indicating that no strand-displacement primer extension occurred via the synthesis of unnatural-natural mispairs after gap filling. Interestingly, the efficiency of Pol *β* for incorporating the unnatural nucleotide for gap filling was generally higher than that for primer extension (Figs 2D and 3D), which might be attributed to an enhancement of the binding affinity of Pol *β* to the one-nucleotide gapped dsDNA by its lyase domain [31, 33]. Next, we investigated the activity of Pol *β* to perform strand-displacement primer extension after gap filling via the synthesis of a UBP (Fig. 3A). The dsDNA substrate with a 1-nt gap was mixed with 10 μM each of dNTPs, 10 μM of one of the unnatural nucleoside triphosphates or none, and 100 nM Pol *β* in 1× Pol *β* reaction buffer and incubated at 37°C for 30 min. As shown in Fig. 3E–G, the primer was extended to a varying length after the synthesis of one of the correctly pairing UBPs, indicating the ability of Pol *β* for strand-displacement primer extension after the gap filling via the synthesis of the UBP. When the dsDNA substrate with a 1-nt gap prepared with T55–TPT3 was used, some degree of primer extension was also observed for the experiment with only dNTPs or with dNTPs and dTPT3TP, dTPT3^Am^TP, or dTPT3^Al^TP. This suggests that Pol *β* is also able to perform strand-displacement primer extension after the gap filling via the synthesis of a dTPT3-natural mispair, a dTPT3–dTPT3 self-pair or one of the analogues of the dTPT3–dTPT3 self-pair. These results suggest the good compatibility of the UBPs with the eukaryotic DNA repair machinery, which should be important for stable expansion of the genetic alphabet in eukaryotic cells.

### Steady-state kinetics for Pol *β*-mediated incorporation of natural or unnatural nucleoside triphosphates opposite a natural or unnatural nucleotide in the DNA template

To rigorously characterize the activity of Pol *β* for the synthesis of different UBPs, we carried out steady-state kinetic experiments. Pol *β* was used to extend FAM-labeled primer FAM-P17 by incorporating one of dNTPs, dTPT3TP, dNaMTP, or dCNMOTP opposite a dT, dNaM, dCNMO, or dTPT3 in template T55–T, T55–NaM, T55–CNMO, or T55–TPT3 (Supplementary Table S1), respectively. Steady-state kinetic constants were calculated by fitting the data to the Michaelis–Menten equation, and the fitted curves were presented in Supplementary Fig. S5. As shown in Table 1, the *K*_m_ values for the incorporation of the unnatural nucleoside triphosphates opposite their complementary nucleotides in the templates were comparable with or even lower than that for the incorporation of dATP opposite a dT in the template. Similarly, comparable *K*_m_ values for T4 DNA polymerase D219A and phi29 DNA polymerase D12A-mediated incorporation of dTPT3TP opposite a dNaM in the template and incorporation of dTTP opposite a dA or dATP opposite a dT in the template were observed in our previous work [22, 34]. Although the catalytic efficiencies, as reflected by the *k*_cat_/*K*_m_ values, of Pol *β* for the synthesis of dNaM–dTPT3, dCNMO–dTPT3, dTPT3–dNaM, and dTPT3–dCNMO were 1.86 × 10^2^, 85.70, 8.71 × 10^2^, and 87.90-fold lower than that for the synthesis of dT-dA, they were significantly higher than those for the synthesis of the self-pairs of the unnatural bases and unnatural-natural mispairs (Table 1). No detectable syntheses of the self-pairs of the unnatural bases, except for dTPT3–dTPT3, and unnatural-natural mispairs were observed, even when the concentrations of the nucleoside triphosphate substrates were increased up to 2 mM, and the reaction was incubated for up to 30 min. These results demonstrate that Pol *β* can synthesize the UBPs with good specificity, with the following order of synthesis efficiencies for different base pairs: dCNMO–dTPT3 ≈ dTPT3–dCNMO > dNaM–dTPT3 > dTPT3–dNaM > dTPT3–dTPT3 > other tested base pairs. Notably, the efficiencies for the synthesis of dCNMO–dTPT3 and dTPT3–dCNMO are significantly higher than those for the synthesis of dNaM–dTPT3 and dTPT3–dNaM, further suggesting that dCNMO–dTPT3 and dTPT3–dCNMO are UBPs better recognized by Pol *β* and might be better to be used for the construction of eukaryotes with an expanded genetic alphabet. Consistent with this, dCNMO–dTPT3 was found to be an optimal UBP for the storage and retrieval of increased genetic information in *E. coli* cells [12]. Notably, while the results described above demonstrated the capability of Pol *β* to synthesize and extend the UBPs with reasonable efficiency and specificity, the synthesis efficiencies of the UBPs by Pol *β* are significantly lower than those by some prokaryotic DNA polymerases, such as KF or T4 DNA polymerase D219A and phi29 DNA polymerase [29] (Table 1). This is similar with the previously reported result for the synthesis efficiencies of some earlier UBPs, such as d5SICS-dMMO2, by Pol *β* and some prokaryotic DNA polymerases [20]. This divergence may arise from the structural differences of Pol *β* and the tested prokaryotic DNA polymerases, since Pol *β* is an X-family DNA polymerase, and the tested prokaryotic DNA polymerases are mainly from families A and B. Additionally, Pol *β*’s primary role in BER prioritizes the specificity over the efficiency for nucleotide incorporation, and this functional constraint may also limit its efficiency for the synthesis of the UBPs [18, 19]. In the future, efforts should be made on investigating the activities of the major replicative eukaryotic DNA polymerases for UBP synthesis.

**Table 1.** Steady-state kinetic constants for Pol *β*-mediated incorporation of a natural or unnatural nucleoside triphosphate (dYTP) opposite a natural or unnatural nucleotide (X) in the DNA template

5′-FAM-CGTATGTTGTGTGGACT				
3′-CCGAAGCATACAACACACCTGAXCTCTCTGGTATTGTTAAAGTGTGTCCTTTGTC				
X	Y	*K*_m_ (μM)	*k*_cat_ (min^−1^)	*k*_cat_/*K*_m_ (M^−1^·min^−1^)
T	A	28.74 ± 6.40	23.60 ± 1.98	8.21 × 10^5^
NaM	TPT3	43.07 ± 6.34	0.19 ± 0.010	4.41 × 10^3^
	NaM	n.d.^a^	n.d.^a^	n.d.^a^
	CNMO	n.d.^a^	n.d.^a^	n.d.^a^
	A	n.d.^a^	n.d.^a^	n.d.^a^
	T	n.d.^a^	n.d.^a^	n.d.^a^
	C	n.d.^a^	n.d.^a^	n.d.^a^
	G	n.d.^a^	n.d.^a^	n.d.^a^
CNMO	TPT3	21.91 ± 1.90	0.21 ± 0.0054	9.58 × 10^3^
	CNMO	n.d.^a^	n.d.^a^	n.d.^a^
	NaM	n.d.^a^	n.d.^a^	n.d.^a^
	A	n.d.^a^	n.d.^a^	n.d.^a^
	T	n.d.^a^	n.d.^a^	n.d.^a^
	C	n.d.^a^	n.d.^a^	n.d.^a^
	G	n.d.^a^	n.d.^a^	n.d.^a^
TPT3	NaM	50.89 ± 7.55	0.048 ± 0.0020	9.43 × 10^2^
	CNMO	11.78 ± 2.41	0.11 ± 0.0058	9.34 × 10^3^
	TPT3	186.70 ± 45.12	0.029 ± 0.0014	1.55 × 10^2^
	A	n.d.^a^	n.d.^a^	n.d.^a^
	T	n.d.^a^	n.d.^a^	n.d.^a^
	C	n.d.^a^	n.d.^a^	n.d.^a^
	G	n.d.^a^	n.d.^a^	n.d.^a^

a n.d.: Rates too slow to determine k_cat_ and K_*m*_ independently.

### Pol *β*-mediated sequencing of DNA oligonucleotides containing one unnatural base

**Figure 4.**
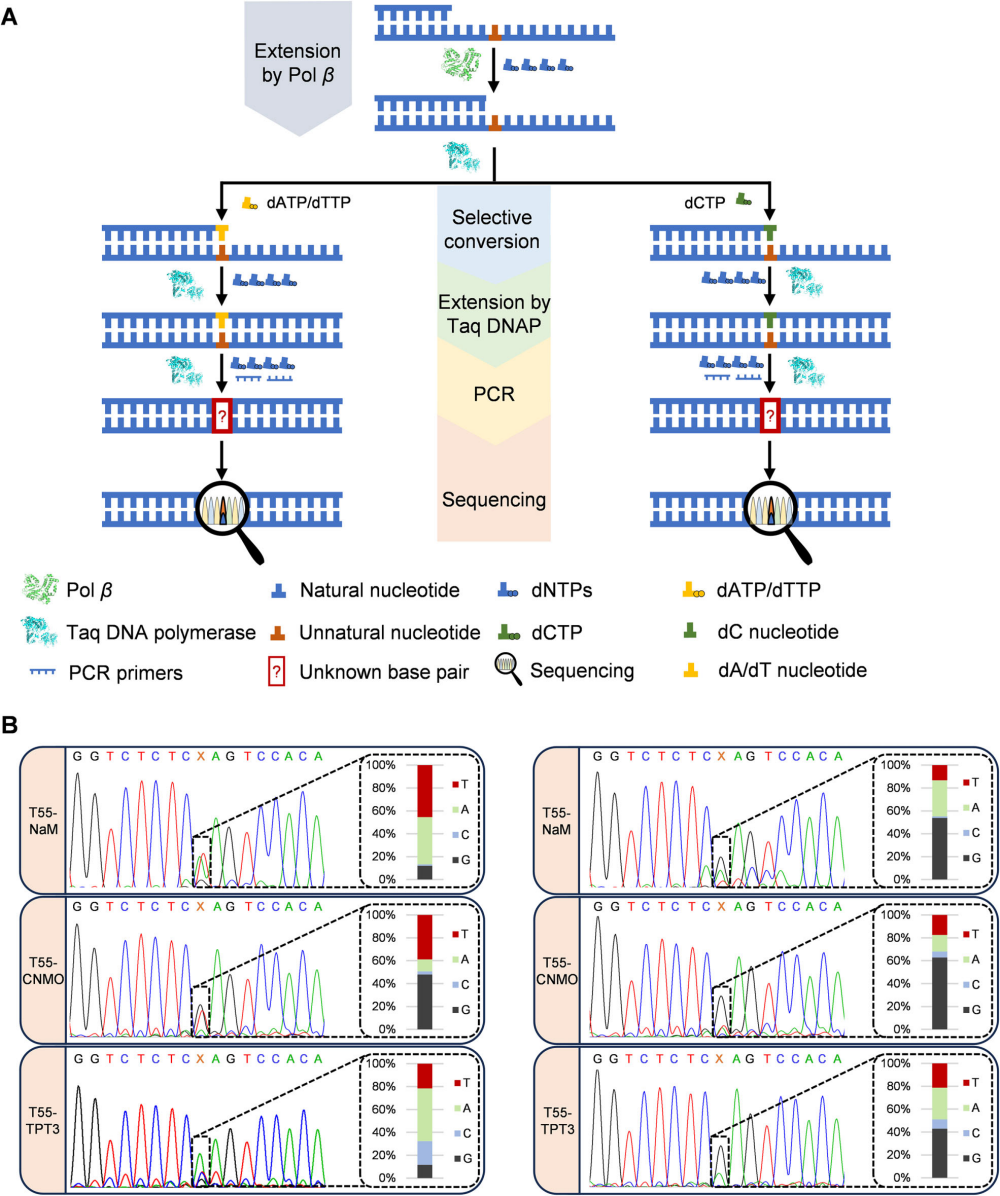
Pol *β*-mediated sequencing of ssDNA oligonucleotides containing a single unnatural base. (**A**) Scheme for the sequencing of ssDNA oligonucleotides containing a single unnatural base with Pol *β*. (**B**) Sanger sequencing results of the dsDNA oligonucleotides generated from ssDNA oligonucleotides containing a dNaM, dCNMO, or dTPT3 (T55–NaM, T55–CNMO, T55–TPT3), and integral analysis for the percentages of the peak areas for different natural nucleotides at the position of the unnatural base. For each case, the sum of the peak areas of all four natural nucleotides is defined as 100%. In the sequence spectra for the selective conversion of T55–NaM with dATP (left) or dCTP (right), X: the position of dNaM in the original ssDNA oligonucleotide; in the sequence spectra for the selective conversion of T55–CNMO with dATP (left) or dCTP (right), X: the position of dCNMO in the original ssDNA oligonucleotide; in the sequence spectra for the selective conversion of T55–TPT3 with dTTP (left) or dCTP (right), X: the position of dTPT3 in the original ssDNA oligonucleotide.

Previous studies have highlighted the significant potential of the UBPs across a spectrum of applications, particularly those in synthetic biology, biotechnology and biomedicine [35–38]. However, there remain significant challenges in sequencing DNAs containing UBPs, which has limited the development and application of UBPs. Taking advantage of the high specificity of Pol *β* for the synthesis of UBPs dNaM–dTPT3 and dCNMO–dTPT3, we attempted to establish a facile method for sequencing DNAs containing unnatural bases dNaM, dCNMO, and dTPT3, which is based on stalled primer extension mediated by Pol *β* and Taq DNA polymerase-mediated selective conversion of an unnatural nucleotide into two different natural nucleotides in parallel and further primer extension (Fig. 4A). In this method, first, a primer is annealed to the DNA oligonucleotide containing an unnatural base to be sequenced and subjected to Pol *β*-mediated primer extension with only dNTPs but no unnatural nucleoside triphosphate. Due to the good specificity of Pol *β* for UBP synthesis, the primer extension is stalled before the position opposite the unnatural nucleotide. Then the primer/template complex is purified to remove the remaining dNTPs and divided into two aliquots for selective conversion of the unnatural nucleotide into different natural ones in parallel. Our earlier investigation revealed that Taq DNA polymerase is able to synthesize unnatural–natural mispairs with good efficiency under optimized reaction conditions [28] (Supplementary Fig. S6A), so it is used for the selective conversion reactions, in one of which the primer is extended with only dATP or dTTP to incorporate a dA or dT opposite the unnatural nucleotide, and in the other the primer is extended with only dCTP to incorporate a dC opposite the unnatural nucleotide. Subsequently, the primer in each reaction is extended to the full length by the addition of the other three dNTPs and further incubation. Then the produced two dsDNAs are PCR amplified with dNTPs and subjected to Sanger sequencing or deep sequencing. By comparing the sequencing results of the two dsDNAs, we should be able to identify the position of the unnatural base, where the percentages of different natural bases (reflected either by the peak areas in the sequence spectra for Sanger sequencing or by the sequence percentages for deep sequencing) should be different for the two dsDNAs, due to the selective conversion of the unnatural nucleotide with different natural nucleoside triphosphates.

To verify the design of this method, we first carried out experiments to test the stalled primer extension mediated by Pol *β* and Taq DNA polymerase-mediated selective conversion of the unnatural nucleotide into different natural nucleotides and further primer extension with different unnatural base-containing DNA templates. The products of different steps were subjected to gel analysis. 5′-FAM-labeled primer FAM-P24-1 was annealed to DNA template T71(TXA)-NaM, T71(CXG)-NaM, T79-NaM, or LIB-NaM; 5′-FAM-labeled primer FAM-P24-2 was annealed to DNA template T55–NaM, T55–CNMO, or T55–TPT3; 5′-FAM-labeled primer FAM-P24-3 was annealed to DNA template T70(TXC)-NaM; 5′-FAM-labeled primer FAM-P24-4 was annealed to DNA template T70(AXT)-NaM; and 5′-FAM-labeled primer FAM-P24-5 was annealed to DNA template T40(GXC)-NaM (Supplementary Table S1), respectively. Then the primer/template complex was mixed with 10 μM each of dNTPs and 300 nM Pol *β* in 1× Pol *β* reaction buffer, without the addition of any unnatural triphosphate, and incubated at 37°C for 20 min. The products were analyzed with denaturing PAGE gels. As shown in Supplementary Fig. S6 B-D, for all the templates with different sequences, in the absence of the unnatural nucleoside triphosphate, the Pol *β*-mediated primer extension was stalled before the position opposite the unnatural nucleotide, evidenced by a sharp band much lower than that for the full-length primer extension product on the gel. Next, we tested the efficiencies for the selective conversion of each of the unnatural nucleotides to different natural nucleotides by incorporating different natural nucleotides opposite an unnatural nucleotide in the DNA template with Taq DNA polymerase. FAM-labeled primer P24-2 was annealed to the DNA template containing a dNaM, dCNMO, or dTPT3 (T55–NaM, T55–CNMO, or T55–TPT3) (Supplementary Table S1) and extended with dATP, dTTP, dCTP, or dGTP by Taq DNA polymerase. The products were analyzed with denaturing PAGE gels. As shown in Supplementary Fig. S6A, for all of the tested templates, the incorporations of dATP and dTTP opposite the unnatural nucleotide were the most efficient, the incorporation of dCTP was moderately efficient, and the incorporation of dGTP was the least efficient. Therefore, only dATP, dTTP, and dCTP were chosen for the selective conversion of the unnatural nucleotides. Subsequently, each of the purified products of the stalled primer extension mediated by Pol *β* was divided into two aliquots. One aliquot was subjected to the selective conversion with dATP (for template T55–NaM or T55–CNMO) or dTTP (for template T55–TPT3), and the other was subjected to the selective conversion with dCTP. All the selective conversion reactions were carried out by mixing the product of the stalled primer extension with the chosen dNTP and Taq DNA polymerase and incubating at 68°C for 30 min. Then for each reaction, the primer was further extended to the full length by the addition of the other three dNTPs and incubation at 68°C for 5 min. The products were analyzed with denaturing PAGE gels. As shown in Supplementary Fig. S6B–D, for all cases, after the selective conversion, all primers were efficiently extended to the full length. Clearly, all the steps tested above were efficient enough for the establishment of the sequencing method.

The dsDNA products of the selective conversion and further primer extension were then PCR amplified and subjected to Sanger sequencing. As shown in Fig. 4B, in the sequence spectra from Sanger sequencing, overlapping peaks for different natural bases were observed at the positions of the unnatural bases. For each template, the ratios of the peak areas for different natural bases are significantly different between the sequences from the selective conversions with dATP/dTTP and dCTP. Specifically, for the templates containing dNaM or dCNMO, the selective conversion was carried out by the incorporation of dATP and dCTP opposite the unnatural nucleotide in the template. In the sequences from the selective conversion with dATP for templates T55–NaM and T55–CNMO, the peak areas for dT account for, respectively, 45.48% and 38.78%, of the sums of the peak areas for all four natural bases at the position of the unnatural base, and the peak areas for dG account for, respectively, 12.06% and 47.92% (Supplementary Table S2). In the sequences from the selective conversion with dCTP for these templates, the percentages of the peak areas for dT decrease to, respectively, 13.40% and 17.60%, and the percentages of the peak areas for dG increase to, respectively, 53.92% and 62.80%. For the template containing dTPT3, the selective conversion was carried out by the incorporation of dTTP and dCTP opposite the unnatural nucleotide in the template. In the sequence from the selective conversion with dTTP for template T55–TPT3, the peak area for dA accounts for 46.03% of the sum of the peak areas for all four natural bases at the position of the unnatural base, and the peak area for dG accounts for 11.51%. In the sequence from the selective conversion with dCTP for this template, the percentage of the peak area for dA decreases to 27.64%, and the percentage of the peak area for dG increases to 42.84%. These results are generally in line with the expectation and suggest the effectiveness of our method for identifying the presence of an unnatural base and locating it in a DNA sequence.

To evaluate the influence of the upstream and downstream natural bases adjacent to the unnatural base on the performance of this method, we further carried out experiments to sequence oligonucleotides containing different natural bases flanking an unnatural base (dNaM) (Supplementary Fig. S7A). For each of the tested oligonucleotides, significant difference is observed in the ratios of the peak areas for different natural bases at the position of the unnatural base between the sequences from the selective conversions with dATP and dCTP (Supplementary Fig. S7B). For the sequences from the selective conversion with dATP, the peak areas for dT account for 83.86%, 95.69%, 64.20%, 86.41%, and 76.42% of the sums of the peak areas for all four natural bases at the position of the unnatural base, and the peak areas for dG account for 0.37%, 2.34%, 10.80%, 1.58%, and 7.22%; for the sequences from the selective conversion with dCTP, the percentages of the peak areas for dT decrease to 46.13%, 32.99%, 27.23%, 51.82%, and 48.53%, and the percentages of the peak areas for dG increase to 32.58%, 59.28%, 64.37%, 24.46%, and 23.76% (Supplementary Table S2). It is worth noting that, for sequences containing a dC flanking the unnatural base, the selective conversion with dCTP was generally more efficient, and the efficiencies of the selective conversion with dATP are also different for different oligonucleotides. However, these differences between the efficiencies of the selective conversion with dATP or dCTP for different oligonucleotides have no effect on the effective identification of the position of the unnatural base, suggesting the good applicability of our method for sequencing unnatural base-containing oligonucleotides with diverse sequences flanking the unnatural base.

It is worth noting that, the ratio of the peak areas for different natural bases at the position of the unnatural base in the sequence spectrum varies along with both the type of the unnatural base and the sequence context around the unnatural base, suggesting that these two factors should have some effect on the processes of the selective conversion, subsequent primer extension, or/and PCR amplification. Moreover, in some sequence spectra, at the position of the unnatural base, relatively high percentages of the peak areas for natural bases that are not resulted from the selective conversion are observed. Presumably, this is mainly due to the fact that in our current method, the original DNA template containing the unnatural base was not removed in the whole process and thus was amplified during the final PCR amplification of the primer extension product with dNTPs, in which the unnatural base in the DNA template was converted into different natural bases with a ratio related to the preference for unnatural-natural conversion of Taq DNA polymerase used in PCR [28]. Additionally, incomplete selective conversion might have also contributed to this. Nevertheless, these facts do not compromise the effectiveness of our method for the identification and location of an unnatural base in a DNA sequence, which is mainly based on the appearance of the overlapping peaks for different natural bases at the position of the unnatural base and the difference between their area ratios from different selective conversions. We also tested whether better sequencing results could be obtained by removing the original DNA template containing the unnatural base via 5′ phosphorylation of it with T4 PNK at the beginning and degradation of it with lambda exonuclease after the selective conversion and subsequent primer extension (Supplementary Figs S8A and S9A). As shown in Supplementary Figs S8B and S9B, by doing this, we obtained much cleaner and better sequence spectra, in which the percentage of the peak area for dT/dA increases to 66.80%–100.00%, at the position of the unnatural base for the selective conversion with dATP/dTTP, and in which the percentage of the peak area for dG increases to 44.29%–84.87%, at the position of the unnatural base for the selective conversion with dCTP (Supplementary Table S3). Clearly, in our method, the original DNA template containing the unnatural base can be removed to obtain better sequencing results, if necessary.

### Pol *β*-mediated sequencing of a DNA oligonucleotide containing multiple unnatural bases

**Figure 5.**
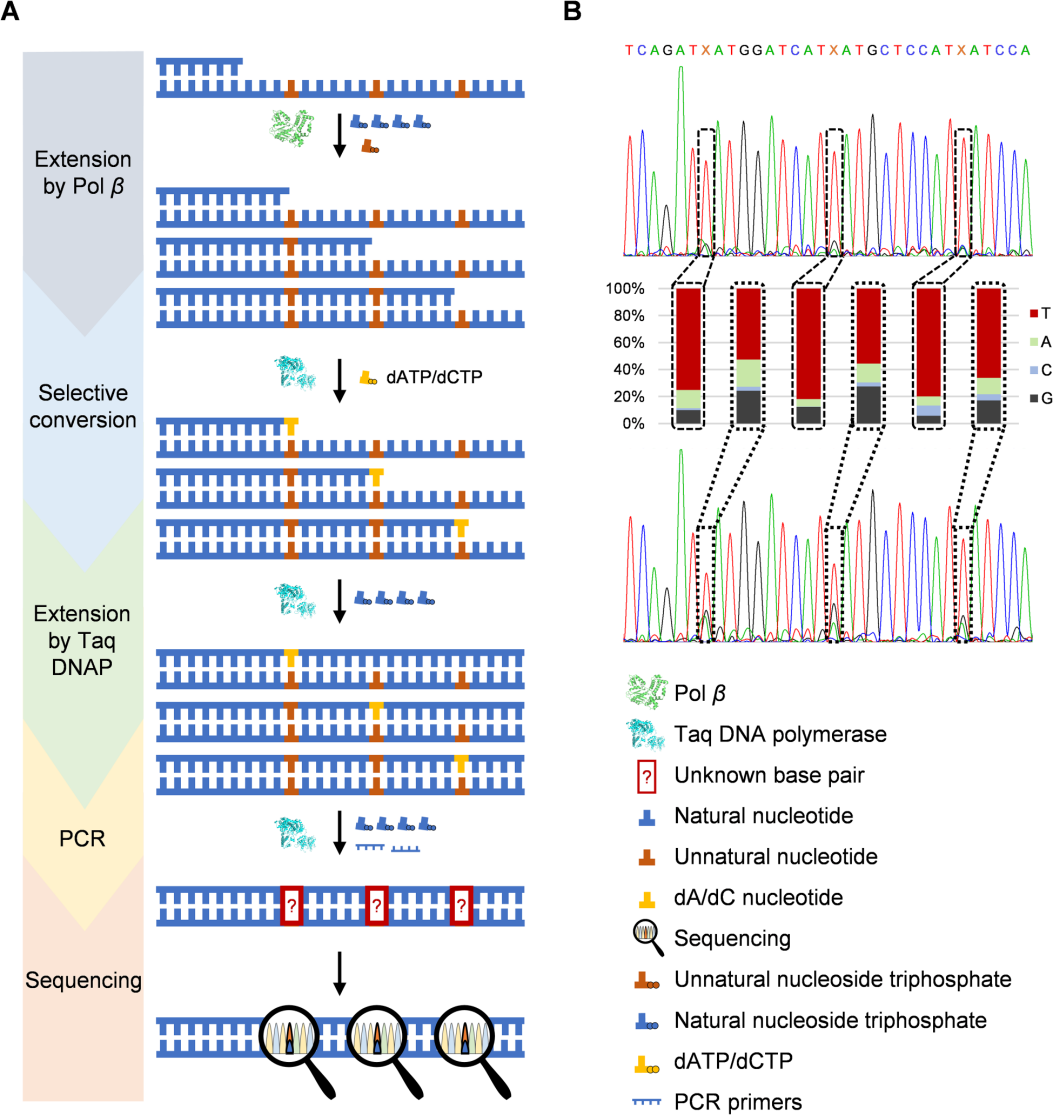
Pol *β*-mediated sequencing of an ssDNA oligonucleotide containing multiple unnatural bases. (**A**) Scheme for the sequencing of an ssDNA oligonucleotide containing multiple unnatural bases. (**B**) Sanger sequencing results of the dsDNA oligonucleotides generated from ssDNA oligonucleotide T79-NaM, which contains three dNaMs, and integral analysis for the percentages of the peak areas for different natural nucleotides at the positions of the unnatural bases. For each case, the sum of the peak areas of all four natural nucleotides is defined as 100%. The selective conversion was done with dATP (upper) or dCTP (lower). X: the position of dNaM in the original ssDNA oligonucleotide.

With the successful sequencing of DNAs containing an unnatural base, we next explored to extend our method to the sequencing of DNAs containing multiple unnatural bases (Fig. 5A). To achieve this goal, we modified our method by doping a low concentration of the corresponding unnatural nucleoside triphosphate into the reaction solution and adjusting the reaction time for the stalled primer extension mediated by Pol *β*, so that the primer extension is partially stalled before the position opposite each unnatural nucleotide, allowing for subsequent partial selective conversion of each unnatural nucleotide into different natural ones in parallel. After the selective conversion, the primer is again further extended to the full length by the addition of the other three dNTPs, and the primer extension product is PCR-amplified with dNTPs. Then the produced dsDNAs were subjected to Sanger sequencing. Hopefully, by comparing the sequencing results from the selective conversion reactions with different natural nucleoside triphosphates, we should be able to identify and locate the unnatural bases in the original DNA sequence, similarly as in the sequencing of DNAs containing a single unnatural base described above.

To assess the feasibility of this modified method, stalled primer extension mediated by Pol *β* was carried out with template T79-NaM, which contains three dNaMs with 9-nt intervals in its sequence. Ten micromolars dTPT3TP was doped into the reaction solution, and the reaction was incubated at 37°C for 30 min. Then Taq DNA polymerase-mediated selective conversion of the dNaM nucleotide with dATP or dCTP and further primer extension was carried out as described above. Gel analysis of the products from different steps clearly suggested the good efficiency of all the steps (Supplementary Fig. S6D). The primer extension products were then PCR amplified and the produced dsDNAs were subjected to Sanger sequencing. As shown in Fig. 5B, overlapping peaks for different natural bases were observed at the positions of all three dNaMs in the sequence spectra from different selective conversions, more clearly in the sequence spectrum from the selective conversion with dCTP. In the sequence spectrum from the selective conversion with dATP, the percentages of the peak areas for dTs at the positions of the three dNaMs are, respectively, 75.23%, 81.91%, and 79.97%, and those for dGs at the positions of the three dNaMs are, respectively, 9.93%, 12.30%, and 5.64% (Supplementary Table S2). In the sequence spectrum from the selective conversion with dCTP, the percentages of the peak areas for dTs at the positions of the three dNaMs decrease to 52.62%, 55.64%, and 66.10%, respectively, and those for dGs increase to 24.19%, 27.49%, and 17.10%, respectively (Supplementary Table S2). From these results, the dNaMs can be successfully identified and located in the DNA template, suggesting the feasibility of our method for sequencing DNAs with multiple unnatural bases. However, due to the partial selective conversion, the difference between the ratios of the peak areas for different natural bases at the position of each unnatural base in the sequence spectra from different selective conversions is reduced, compared to that for the sequencing of DNAs containing a single unnatural base, resulting in greater challenge for sequencing DNAs with more unnatural bases. We also tested whether better sequencing results can be obtained by removing the original DNA template containing the unnatural bases via 5′ phosphorylation of it with T4 PNK at the beginning and degradation of it with lambda exonuclease after the selective conversion and subsequent primer extension (Supplementary Fig. S10A). As shown in Supplementary Fig. S10B, this manipulation leads to some increase in the percentages of the peak areas for dGs at some positions of the unnatural bases in the sequence spectrum from the selective conversion with dCTP, but not too much change in the sequence spectrum from the selective conversion with dATP.

### Pol *β*-mediated sequencing of a random DNA oligonucleotide library containing an unnatural base

**Figure 6.**
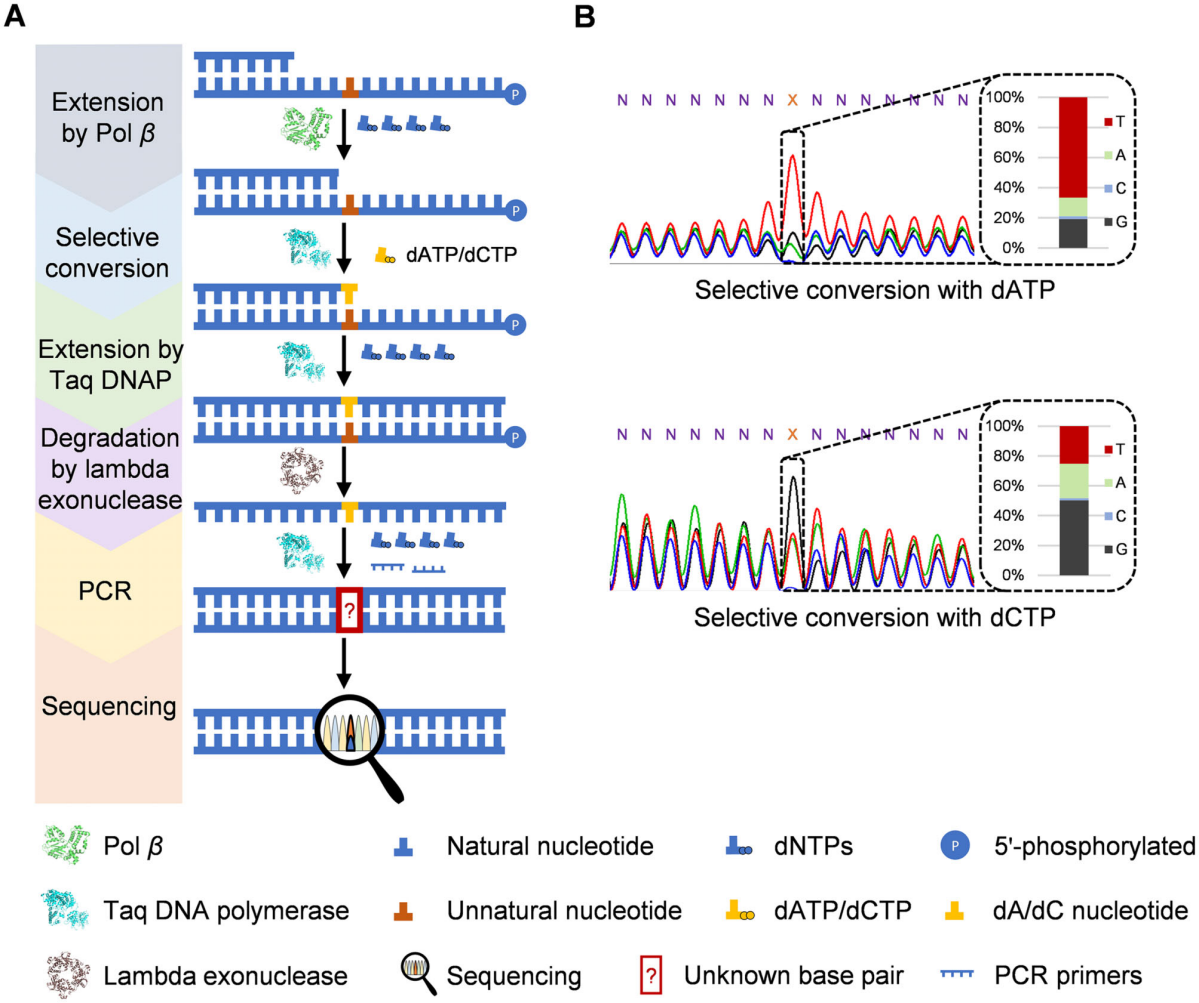
Pol *β*-mediated sequencing of a random ssDNA oligonucleotide library containing a single unnatural base, in which the original ssDNA oligonucleotide library was initially phosphorylated at the 5′ end and removed by lambda exonuclease degradation after the primer extension by Taq DNA polymerase. (**A**) Scheme for the sequencing of a random ssDNA oligonucleotide library containing a single unnatural base. (**B**) Sanger sequencing results of the ssDNA oligonucleotide libraries generated from ssDNA oligonucleotide library LIB–NaM, and integral analysis for the percentages of the peak areas for different natural nucleotides at the position of the unnatural base. For each case, the sum of the peak areas of all four natural nucleotides is defined as 100%. X: the position of dNaM in the original ssDNA oligonucleotide library.

To further explore the universality of our sequencing method, we tested its performance in sequencing a random DNA library containing an unnatural base, exemplified by dNaM. Stalled primer extension mediated by Pol *β* and Taq DNA polymerase-mediated selective conversion of the dNaM nucleotide with dATP or dCTP and further primer extension were carried out as in the sequencing of a DNA template with a single sequence and a dNaM described above. Gel analysis of the products of different steps also revealed the good efficiencies of all the steps (Supplementary Fig. S6D). The products of the selective conversion and further primer extension were again PCR-amplified, and the produced dsDNAs were subjected to Sanger sequencing (Supplementary Fig. S11A). As shown in Supplementary Fig. S11B, overlapping peaks for different natural bases were observed at the position of dNaM in the sequence spectra from different selective conversions. In the sequence spectrum from the selective conversion with dATP, the percentage of the peak area for dT is 79.82%, and that for dG is 9.92%. In the sequence spectrum from the selective conversion with dCTP, the percentage of the peak area for dT decreases to 73.13%, and that for dG increases to 18.93%. Since the original library containing dNaM was not removed, it should be amplified during the PCR amplification of the product of the selective conversion and further primer extension, by which dNaM in the library was converted into different natural bases. This again should be one reason for the high percentages of the peak areas for natural bases that are not resulted from the selective conversion. Surprisingly, a significant increase in the percentage of the peak area for dT, which in principle should be roughly the same as those for other bases, is observed at the 3′ position adjacent to dNaM in the sequence spectra from both selective conversions (Supplementary Fig. S11B). Previous studies have demonstrated that the unnatural bases can affect the amplification bias of the bases adjacent to them during PCR [26, 39], which may explain this unexpected result. Similar as the sequencing experiments described above, to address these issues and obtain better sequencing results, we optimized the method by removing the original DNA library containing a dNaM via 5′ phosphorylation of it with T4 PNK at the beginning and degradation of it with lambda exonuclease after the selective conversion and subsequent primer extension (Fig. 6A). As shown in Fig. 6B, in the sequence spectrum from the selective conversion with dCTP, this optimization leads to a significant increase in the percentage of the peak area for dG at the position of dNaM (from 18.93% to 50.25%), as well as closer percentages of the peak areas for different bases at the 3′ position adjacent to dNaM, which indicate a more reliable sequencing outcome. Based on the protocols and results of all sequencing experiments described above, the method without the removal of the original unnatural base-containing DNA template exhibits advantages in simplicity and speed, while the method with the removal of the original unnatural base-containing DNA template provides clearer and more accurate results.

### Deep sequencing of the natural dsDNAs produced from various DNAs containing unnatural bases for their sequencing mediated by Pol *β*

To more accurately evaluate the established sequencing method for DNAs containing unnatural bases, we further performed deep sequencing of the natural dsDNAs produced in different sequencing experiments described above. Deep sequencing offers several advantages over Sanger sequencing, including higher throughput, increased accuracy, and the ability to obtain the sequences of all DNA oligonucleotides in complex mixtures of a great number of DNA oligonucleotides [40]. For each natural dsDNA sample, over 1945 sequences were achieved, allowing for robust statistical analysis of the sequence composition of each sample and comprehensive evaluation of the selective conversion of the unnatural bases. As shown in Supplementary Fig. S12, deep sequencing data revealed distinct percentages of different natural bases at each of the positions of the unnatural bases in the oligonucleotides in the samples, indicating the successful conversion of the unnatural bases into combinations of natural ones. For the DNA templates containing a single unnatural base, the percentages of dG at the position of the unnatural base are significantly elevated in the samples from the selective conversion with dCTP, with values ranging from 22.2% to 74.08%, compared to that in the samples from the selective conversion with dATP/dTTP, with values ranging from 4.28% to 61.86% (Supplementary Fig. S12A and B, and Supplementary Table S4). Similarly, for the DNA template containing multiple unnatural bases, the percentages of dGs at the positions of the unnatural bases are, respectively, 9.32%, 6.53%, and 5.13% in the sample from the selective conversion with dATP, and increase to, respectively, 18.78%, 21.38%, and 10.70% in the sample from the selective conversion with dCTP (Supplementary Fig. S12C). These results highlight the method’s ability to effectively convert and accurately identify and locate the unnatural bases in different sequence contexts. For the random DNA library containing an unnatural base, the deep sequencing data further demonstrated the robustness of our method. Despite the presence of diverse sequence contexts around the unnatural base and potential amplification bias for different bases during PCR, the selective conversions of the unnatural base with dATP or dCTP were effective. The percentage of dG at the position of the unnatural base is 29.10% in the sample from the selective conversion with dATP and increases to 56.40% in the sample from the selective conversion with dCTP, clearly indicating the location of the unnatural base (Supplementary Fig. S12D and E). These results underscore the method’s capacity to analyze complex samples to provide reliable and accurate sequencing data, further suggesting its broad aplication scope.

## Conclusions

In this study, we explored the activity of Pol *β* for the synthesis of UBPs, specifically dNaM–dTPT3, dCNMO–dTPT3, and their functionalized derivatives and on this basis, developed a new sequencing method for DNAs containing these UBPs. The primer extension assay and steady-state kinetic experiments revealed that Pol *β* could synthesize and extend the tested UBPs with good efficiency and specificity, and the ability of Pol *β* for mediating gap filling via the synthesis of a UBP and subsequent strand-displacement primer extension was also demonstrated. These results indicate the tolerance of the eukaryotic DNA replication machinery against these UBPs, suggesting the possibility of constructing eukaryotic cells with an expanded genetic alphabet, which may find broad use in synthetic biology, biotechnology, and biomedicine. To fully assess the applicability of the UBPs to the eukaryotic systems, efforts should be made to investigate the recognition of the UBPs by other major eukaryotic DNA polymerases, such as Pol *δ* and *ε*, in the future. Based on the recognition of the UBPs by Pol *β*, we established a sequencing method for the DNAs containing unnatural bases, which involves stalled primer extension mediated by Pol *β*, selective conversion of an unnatural nucleotide into two different natural nucleotides in parallel and further primer extension mediated by Taq DNA polymerase, and Sanger or deep sequencing of the produced natural DNAs. This method overcomes the limitations of previous approaches and enables robust detection of one or multiple unnatural bases in diverse sequence contexts, from single sequences to random libraries, which suggests its universality and potential for applications involving high-throughput sequencing, such as systematic evolution of ligands by exponential enrichment (SELEX) and DNA data storage. Compared with other reported methods for sequencing DNAs containing unnatural bases, specifically dNaM, dCNMO, dTPT3, and their functionalized derivatives, such as direct Sanger sequencing, nanopore sequencing, and sequencing with a bridge base, this method has several advantages, including the ability of directly sequencing DNAs containing multiple unnatural bases, less reliability on the sequence adjacent to the unnatural bases, universality for different unnatural bases, and elimination of the need for a bridge base or modified unnatural nucleoside triphosphates. Besides, with minor modification and proper optimization, this method may also be applicable for the sequencing of DNAs containing UBPs from other groups, such as dP–dZ, dS–dB, and dDs–dPx. These clearly suggest the great potential of this method on advancing the application of the UBPs in various fields.

## Supplementary Material

gkaf1460_Supplemental_File

## Data Availability

The data underlying this article are available in the article and in its online supplementary data.
