# Supplementary material for: Recognition of unnatural base pairs by a eukaryotic DNA polymerase enables universal sequencing of an expanded genetic alphabet
Source: Nucleic Acids Res. 2026 Jan 8;54(1):gkaf1460. doi: 10.1093/nar/gkaf1460 (PMC12781883; doi:10.1093/nar/gkaf1460)
Supplement: gkaf1460_Supplemental_File [file gkaf1460_supplemental_file.pdf]

Supplementary Information

Recognition of unnatural base pairs by a eukaryotic DNA polymerase enables universal sequencing of an expanded genetic alphabet

Hantao Luo†, Yuhui Du†, Leping Sun†, Fangkai Ye, Jiezhao Ma, Xueting Wang, Yaxin Wang and Tingjian Chen*

MOE International Joint Research Laboratory on Synthetic Biology and Medicines, School of Biology and Biological Engineering, South China University of Technology, 510006, Guangzhou, China.

*To whom correspondence should be addressed. Email: chentj@scut.edu.cn

†The first three authors should be regarded as Joint First Authors.

Supplementary Figures

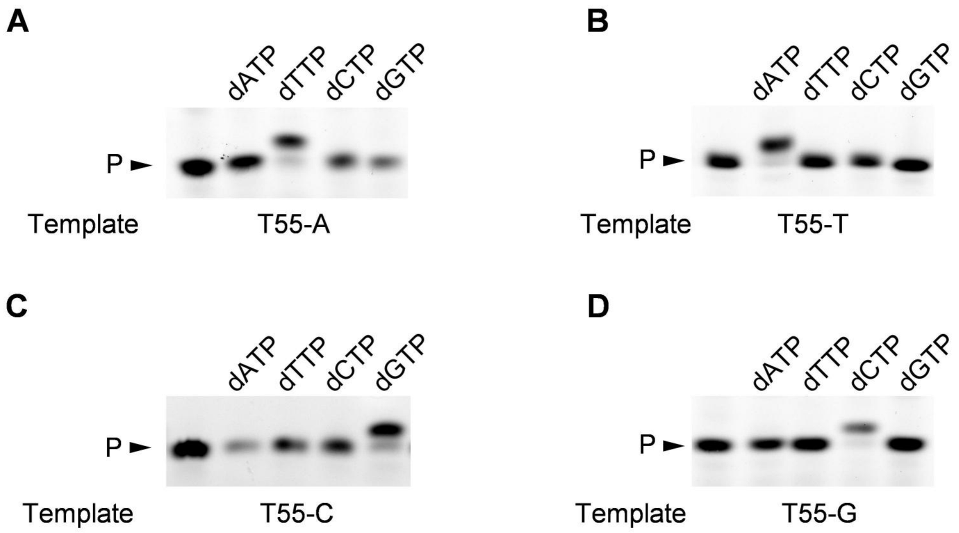

Figure S1. Pol *β*-mediated incorporation of a single natural nucleotide opposite a natural nucleotide in the DNA template. (**A**) Incorporation of a single natural nucleotide opposite a dA in the DNA template (T55-A). (**B**) Incorporation of a single natural nucleotide opposite a dT in the DNA template (T55-T). (**C**) Incorporation of a single natural nucleotide opposite a dC in the DNA template (T55-C). (**D**) Incorporation of a single natural nucleotide opposite a dG in the DNA template (T55-G). For each reaction, 20 nM primer/template complex was mixed with 100 μM of dATP, dTTP, dCTP, or dGTP, 0.5 mg/mL BSA, 7% glycerol, and 100 nM Pol *β* in 1× Pol *β* reaction buffer and incubated at 37 °C for 60 min. The products were analyzed with 20% denaturing PAGE gels supplemented with 8 M urea. P: primer.

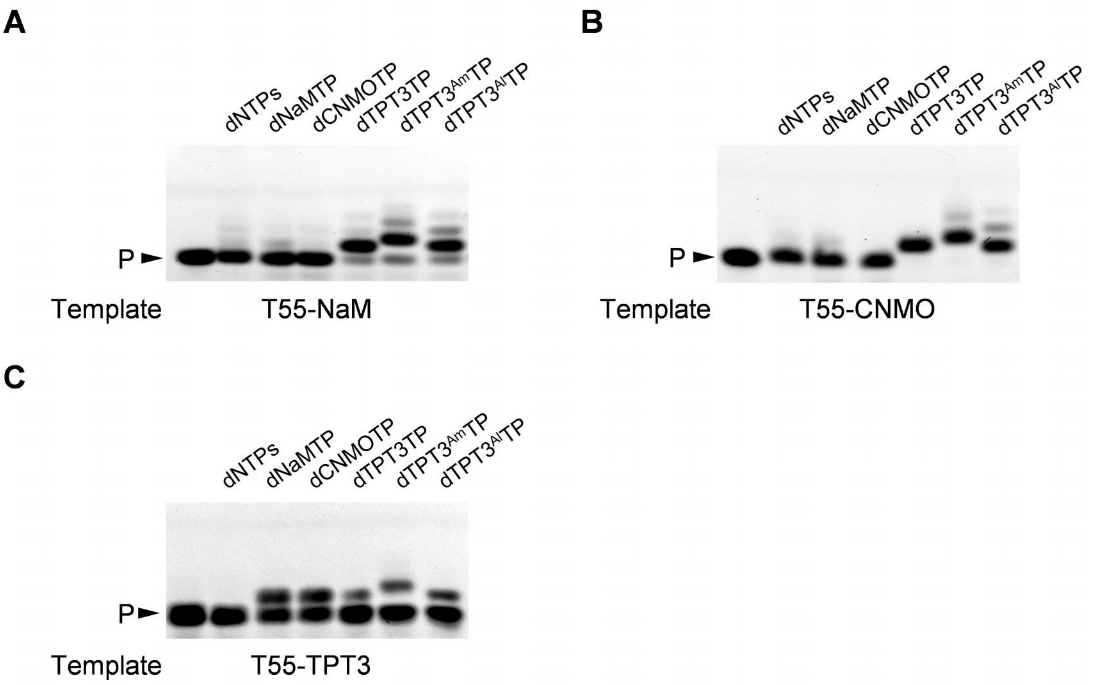

Figure S2. Pol β-mediated incorporation of a single unnatural nucleotide opposite an unnatural nucleotide in the DNA template. (**A**) Incorporation of a single unnatural nucleotide opposite a dNaM in the DNA template (T55-NaM). (**B**) Incorporation of a single unnatural nucleotide opposite a dCNMO in the DNA template (T55-CNMO). (**C**) Incorporation of a single unnatural nucleotide opposite a dTPT3 in the DNA template (T55-TPT3). For each reaction, 20 nM primer/template complex was mixed with 100 μM dNaMTP, dCNMOTP, dTPT3TP, dTPT3^{Am}TP, or dTPT3^{Al}TP, or 100 μM each of dNTPs, 0.5 mg/mL BSA, 7% glycerol, and 100 nM Pol β in 1× Pol β reaction buffer and incubated at 37 °C for 60 min. The products were analyzed with 20% denaturing PAGE gels supplemented with 8 M urea. P: primer.

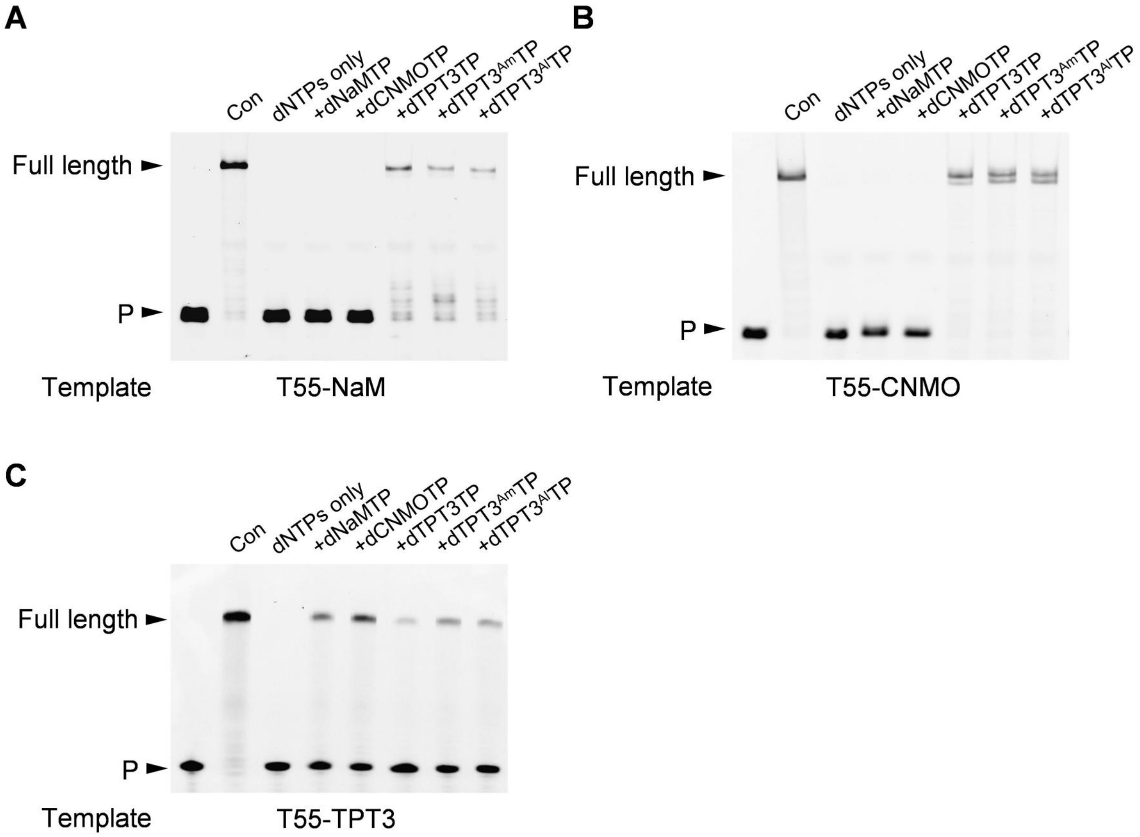

Figure S3. Pol *β*-mediated primer extension with unnatural nucleotide-containing DNA templates and natural and unnatural nucleoside triphosphates**.** (**A**) Pol *β*-mediated primer extension with a DNA template containing a dNaM (T55-NaM) and natural and unnatural nucleoside triphosphates. (**B**) Pol *β*-mediated primer extension with a DNA template containing a dCNMO (T55-CNMO) and natural and unnatural nucleoside triphosphates. (**C**) Pol *β*-mediated primer extension with a DNA template containing a dTPT3 (T55-TPT3) and natural and unnatural nucleoside triphosphates. For each reaction, 20 nM primer/template complex was mixed with 100 μM each of dNTPs, 100 μM dNaMTP, dCNMOTP, dTPT3TP, dTPT3^{Am}TP, dTPT3^{Al}TP, or none, 0.5 mg/mL BSA, 7% glycerol, and 100 nM Pol *β* in 1× Pol *β* reaction buffer and incubated at 37 °C for 60 min. The products were analyzed with 20% denaturing PAGE gels supplemented with 8 M urea. Con: primer extension with a natural DNA template and dNTPs by Pol *β* under the same reaction conditions. P: primer.

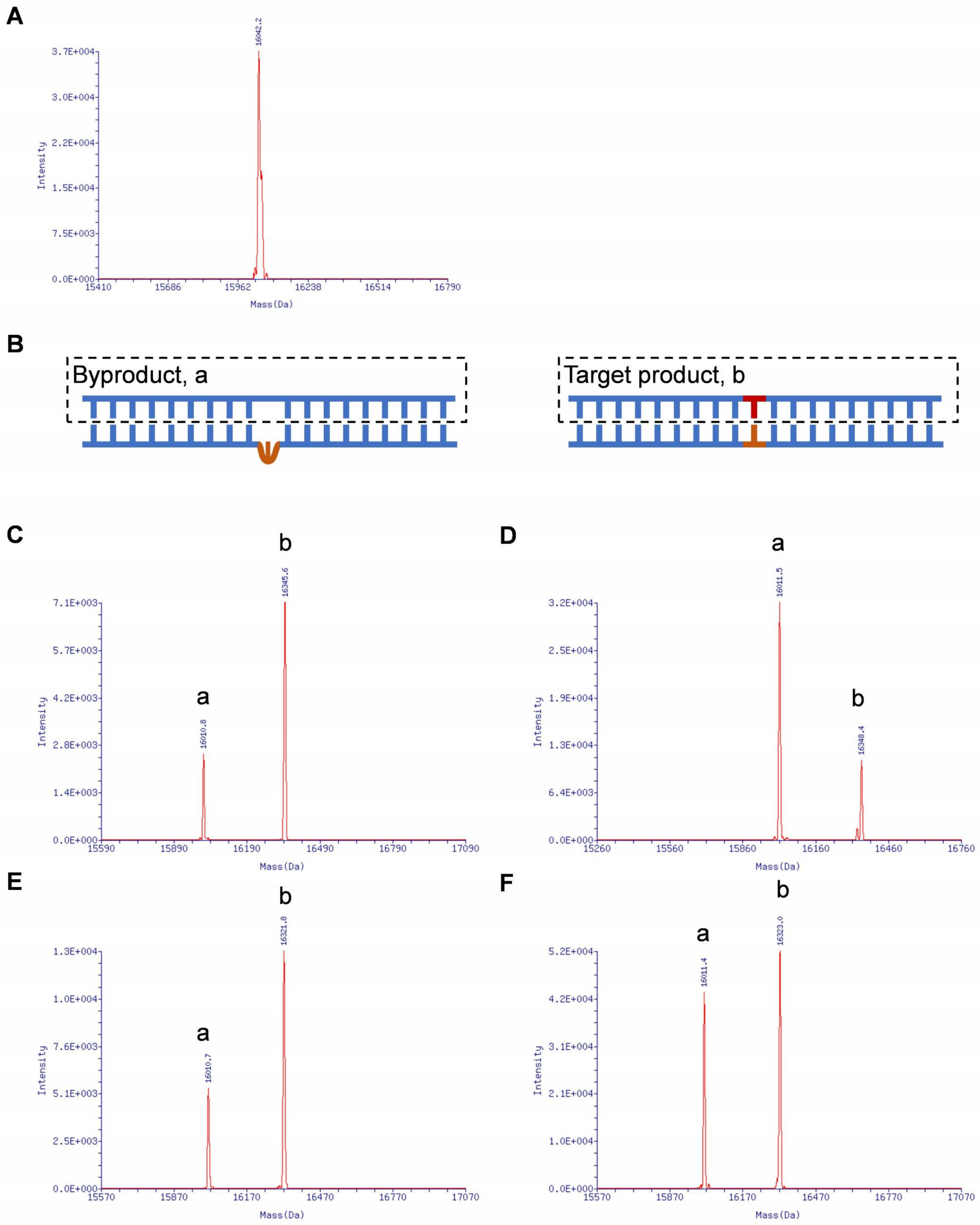

Figure S4. Mass spectrometric analysis of the products of Pol β-mediated primer extension with unnatural nucleotide-containing DNA templates and natural and unnatural nucleoside triphosphates. (**A**) Mass spectrometric analysis of the product of Pol β-mediated primer extension with 10 μM dNaMTP, 10 μM dTPT3TP, and 10 μM each of dNTPs as triphosphate

substrates using a DNA template containing a dNaM (T55-NaM). (**B**) Schematic diagram of byproduct (a) and target product (b) of Pol *β*-mediated primer extension with a DNA template containing a dTPT3 (T55-TPT3). (**C**) Mass spectrometric analysis of the product of Pol *β*-mediated primer extension with 100 μM dNaMTP, 100 μM dTPT3TP, and 100 μM each of dNTPs as triphosphate substrates using a DNA template containing dTPT3 (T55-TPT3). (**D**) Mass spectrometric analysis of the product of Pol *β*-mediated primer extension with 10 μM dNaMTP, 10 μM dTPT3TP, and 10 μM each of dNTPs as triphosphate substrates using a DNA template containing dTPT3 (T55-TPT3). (**E**) Mass spectrometric analysis of the product of Pol *β*-mediated primer extension with 100 μM dCNMOTP, 100 μM dTPT3TP, and 100 μM each of dNTPs as triphosphate substrates using a DNA template containing dTPT3 (T55-TPT3). (**F**) Mass spectrometric analysis of the product of Pol *β*-mediated primer extension with 10 μM dCNMOTP, 10 μM dTPT3TP, and 10 μM each of dNTPs as triphosphate substrates using a DNA template containing dTPT3 (T55-TPT3). For each reaction, 20 nM primer/template complex was mixed with 10 or 100 μM each of dNTPs, 10 or 100 μM dCNMOTP or 10 μM dNaMTP, 10 or 100 μM dTPT3TP, 0.5 mg/mL BSA, 7% glycerol, and 100 nM Pol *β* in 1× Pol *β* reaction buffer and incubated at 37 °C for 60 min. The products were sent to Sangon Biotech Co., Ltd. (Shanghai, China) for Mass spectrometry analysis.

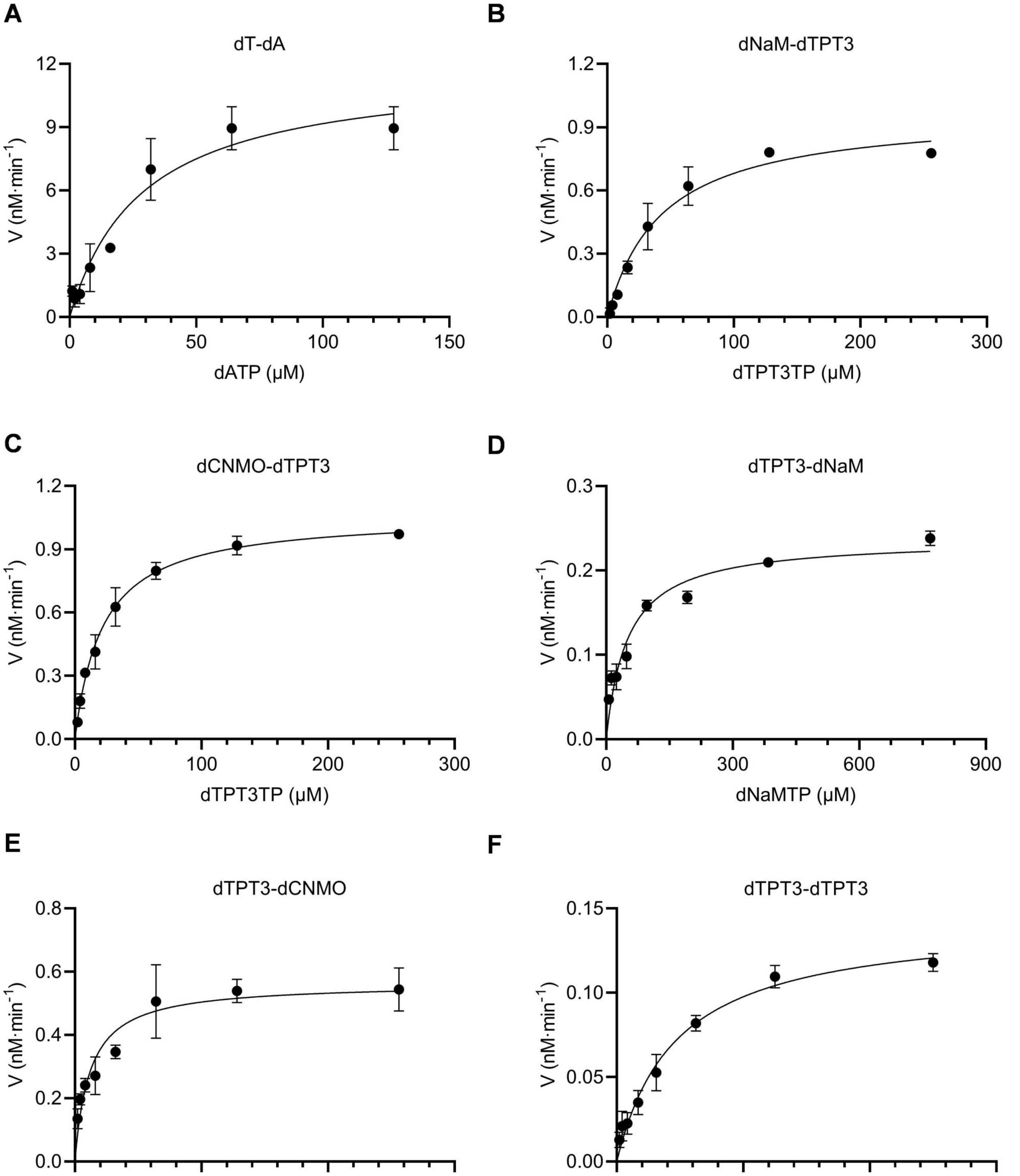

Figure S5. Michaelis-Menten plots for Pol β-mediated incorporation of a natural or unnatural nucleoside triphosphate opposite a natural or unnatural nucleotide in the DNA template. (**A**) Incorporation of dATP opposite a dT in the DNA template. 20 nM primer/template complex FAM-P17/T55-T was mixed with 1-128 μM dATP, 0.5 mg/mL BSA, 7% glycerol, and 0.5 nM Pol β in 1× Pol β reaction buffer and incubated at 37 °C for 20 s. (**B**) Incorporation of

dTPT3TP opposite a dNaM in the DNA template. 20 nM primer/template complex FAM-P17/T55-NaM was mixed with 2-256 μM dTPT3TP, 0.5 mg/mL BSA, 7% glycerol, and 5 nM Pol β in 1× Pol β reaction buffer and incubated at 37 °C for 6 min. (**C**) Incorporation of dTPT3TP opposite a dCNMO in the DNA template. 20 nM primer/template complex FAM-P17/T55-CNMO was mixed with 2-256 μM dTPT3TP, 0.5 mg/mL BSA, 7% glycerol, and 5 nM Pol β in 1× Pol β reaction buffer and incubated at 37 °C for 5 min. (**D**) Incorporation of dNaMTP opposite a dTPT3 in the DNA template. 20 nM primer/template complex FAM-P17/T55-TPT3 was mixed with 6-768 μM dNaMTP, 0.5 mg/mL BSA, 7% glycerol, and 5 nM Pol β in 1× Pol β reaction buffer and incubated at 37 °C for 12 min. (**E**) Incorporation of dCNMOTP opposite a dTPT3 in the DNA template. 20 nM primer/template complex FAM-P17/T55-TPT3 was mixed with 2-256 μM dCNMOTP, 0.5 mg/mL BSA, 7% glycerol, and 5 nM Pol β in 1× Pol β reaction buffer and incubated at 37 °C for 6 min. (**F**) Incorporation of dTPT3TP opposite a dTPT3 in the DNA template. 20 nM primer/template complex FAM-P17/T55-TPT3 was mixed with 8-1024 μM dTPT3TP, 0.5 mg/mL BSA, 7% glycerol, and 5 nM Pol β in 1× Pol β reaction buffer and incubated at 37 °C for 35 min. All the products were analyzed with 20% denaturing PAGE gels supplemented with 8 M urea.

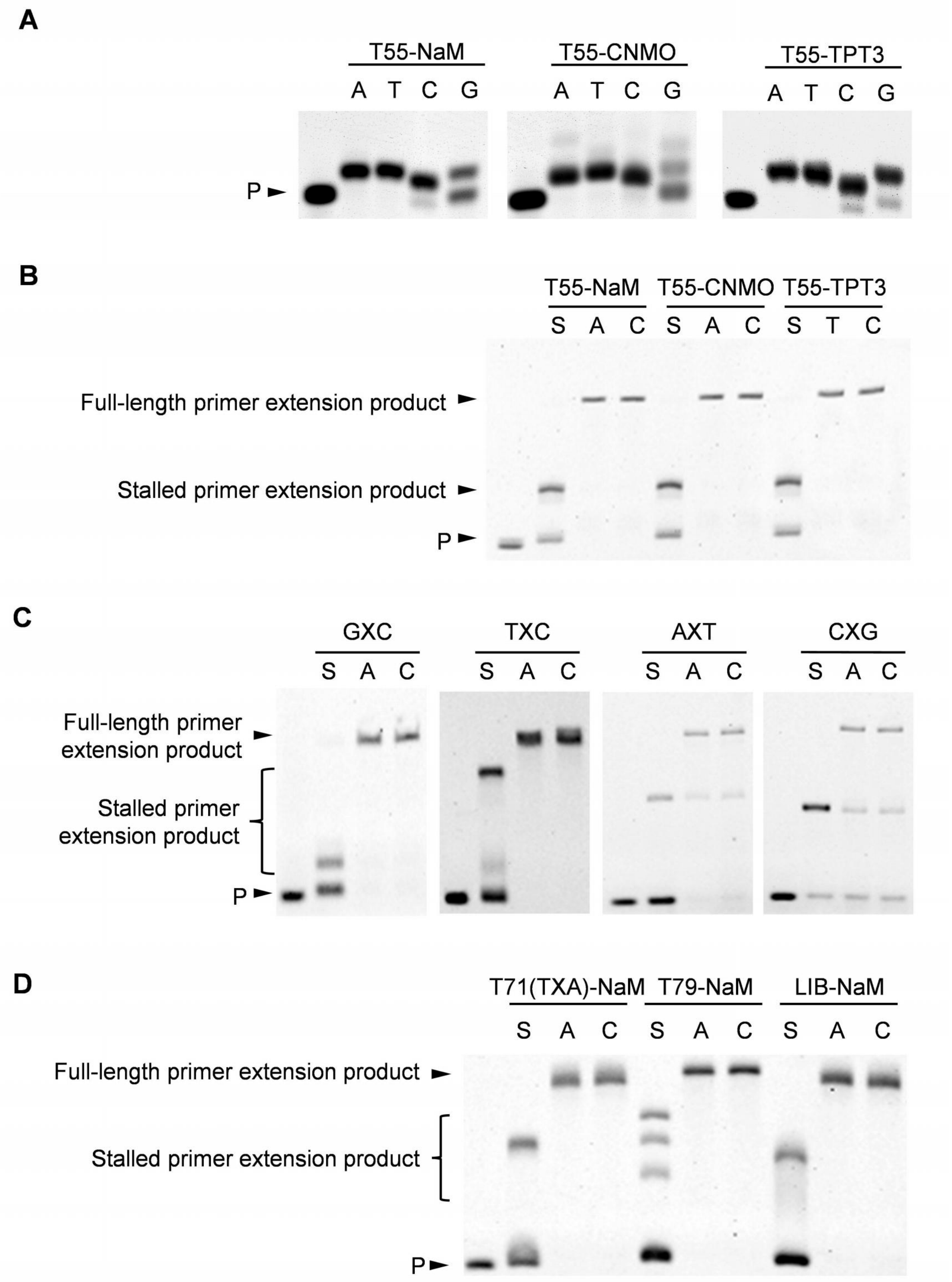

Figure S6. Gel analysis of the intermediate products for the Pol β-mediated sequencing of ssDNA oligonucleotides or an ssDNA oligonucleotide library containing one or multiple unnatural bases. (**A**) Test for the incorporation of different natural nucleotides opposite a dNaM, dCNMO, or dTPT3 in the DNA template by Taq DNA polymerase. For each reaction, 20 nM primer (Cy3-P17)/template (T55-NaM, T55-CNMO, or T55-TPT3) complex was

mixed with 200 μM dATP, dTTP, dCTP, or dGTP and 10 nM Taq DNA polymerase in 1× standard Taq reaction buffer and incubated at 68 °C for 20 min. The products were analyzed with 20% denaturing PAGE gels supplemented with 8 M urea. P: primer. (**B-D**) Products of the stalled primer extension mediated by Pol *β* (S) and further primer extension after selective conversion with dATP (A), dTTP (T), or dCTP (C) mediated by Taq DNA polymerase using different unnatural base-containing templates (GXC: T40(GXC)-NaM; CXG: T71(CXG)-NaM; TXC: T70(TXC)-NaM; AXT: T70(AXT)-NaM). For each reaction of the stalled primer extension mediated by Pol *β*, 0.1 μM primer/template complex was mixed with 10 μM dTPT3TP (only when T79-NaM was used as the template), 10 μM each of dNTPs, 0.5 mg/mL BSA, 7% glycerol, and 300 nM Pol *β* in 1× Pol *β* reaction buffer and incubated at 37 °C for 20 min. An aliquot of the product was saved for gel analysis, and the rest was purified with a Zymo ssDNA/RNA Clean & Concentrator™ kit and subjected to selective conversion and further primer extension by Taq DNA polymerase. For each reaction of the selective conversion and further primer extension by Taq DNA polymerase, 0.4 ng/μL of the stalled primer extension product was mixed with 200 μM dATP (for the selective conversion of dNaM or dCNMO) or dTTP (for the selective conversion of dTPT3), or dCTP, and 10 nM Taq DNA polymerase in 1× standard Taq reaction buffer and incubated at 68 °C for 20 min. Then 200 μM each of the other three dNTPs was added, and the reaction was further incubated at 68 °C for 5 min. All the products were analyzed with 20% denaturing PAGE gels supplemented with 8 M urea.

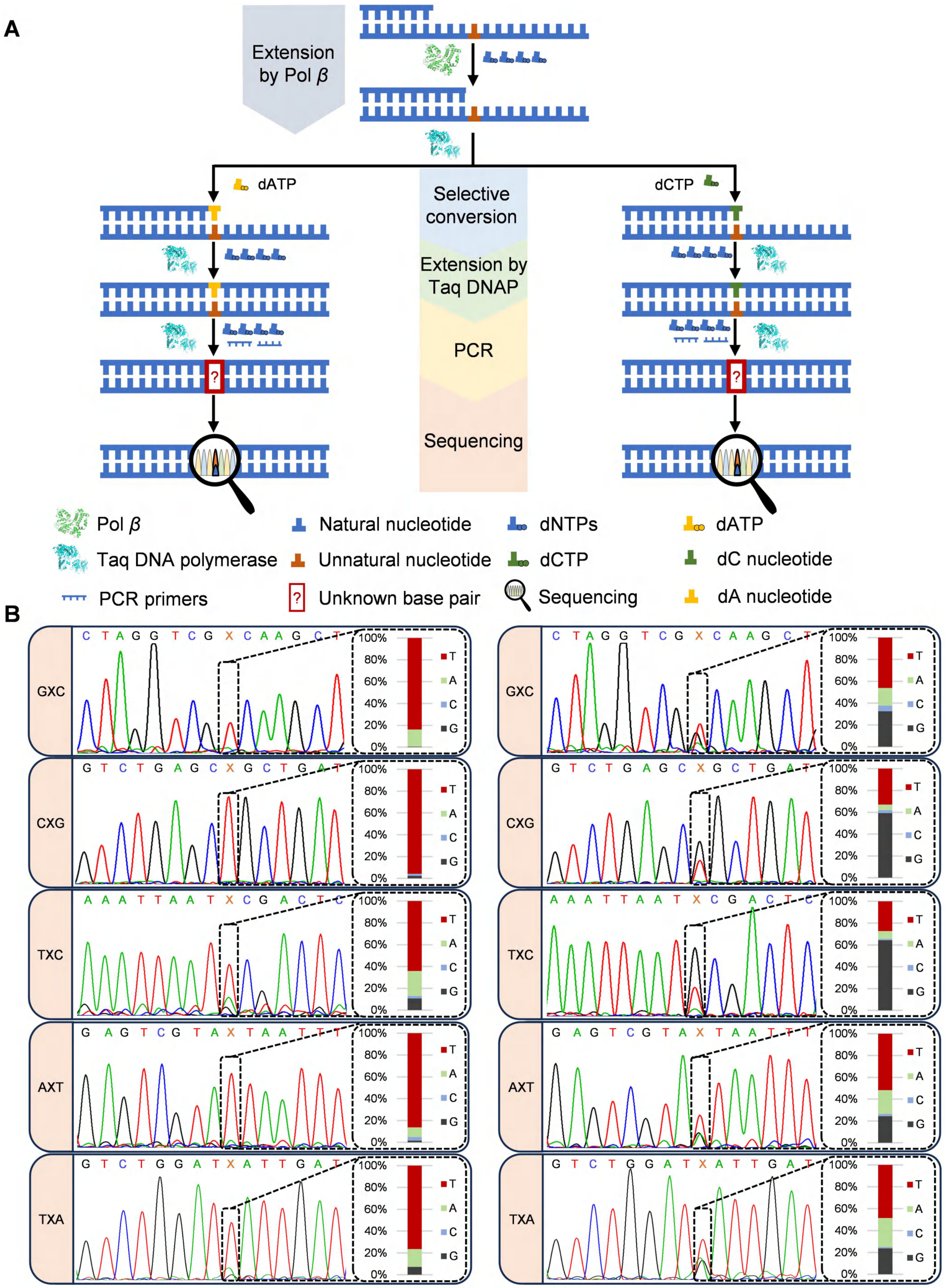

Figure S7. Pol β-mediated sequencing of ssDNA oligonucleotides containing a single

unnatural base and different upstream and downstream natural bases adjacent to the unnatural base, in which the original ssDNA oligonucleotide was not removed by 5' phosphorylation and lambda exonuclease degradation. (**A**) Scheme for the sequencing of ssDNA oligonucleotides containing a single unnatural base and different upstream and downstream natural bases adjacent to the unnatural base. (**B**) Sanger sequencing results of the dsDNA oligonucleotides generated from ssDNA oligonucleotides containing a dNaM and different upstream and downstream natural bases adjacent to the dNaM [GXC: T40(GXC)-NaM; CXG: T71(CXG)-NaM; TXC: T70(TXC)-NaM; AXT: T70(AXT)-NaM; TXA: T71(TXA)-NaM], and integral analysis for the percentages of the peak areas for different natural nucleotides at the position of the dNaM. For each case, the sum of the peak areas of all four natural nucleotides was defined as 100%. The selective conversion for all ssDNA oligonucleotides was done with dATP (left) or dCTP (right). X: the position of dNaM in the original ssDNA oligonucleotide.

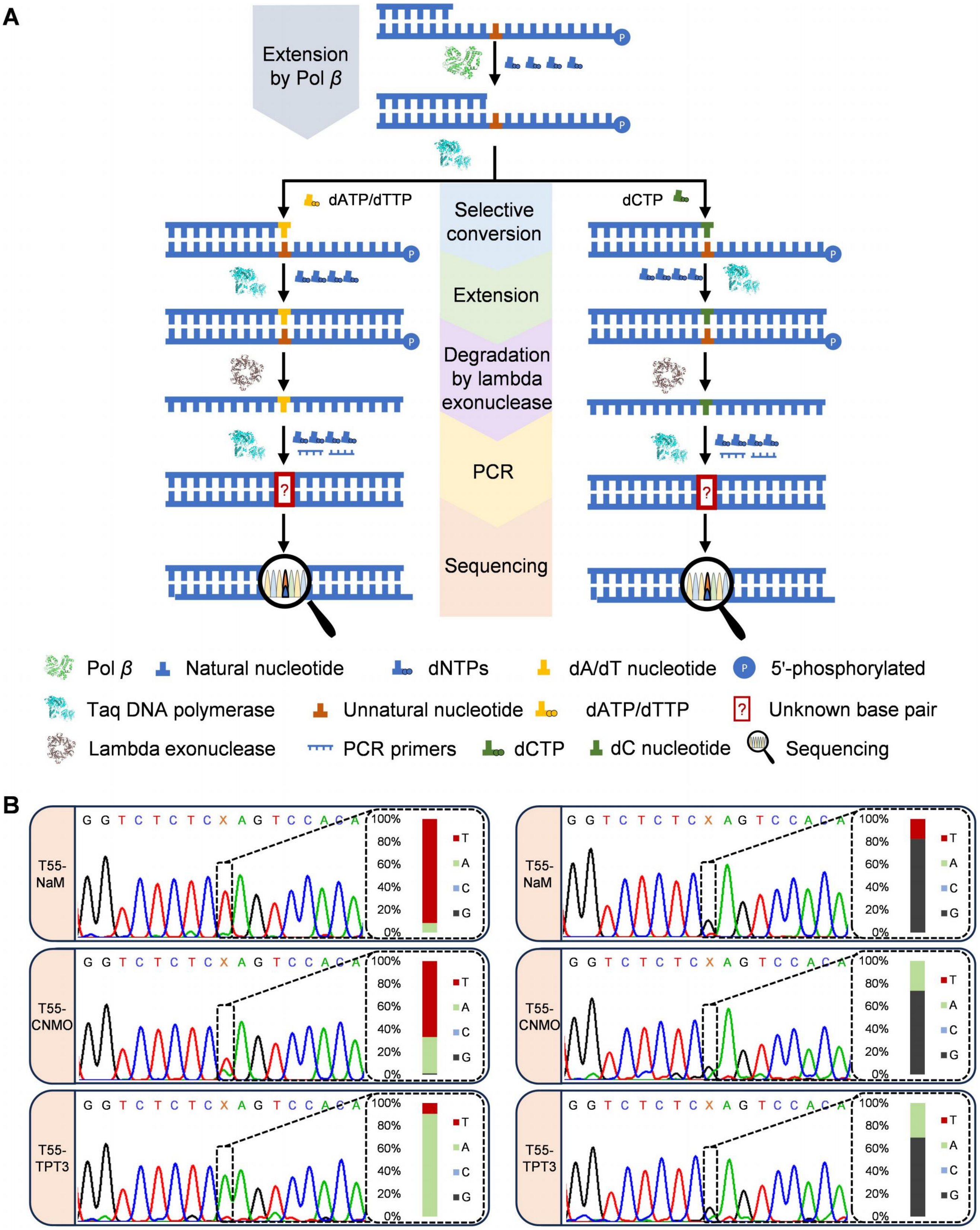

Figure S8. Pol *β*-mediated sequencing of ssDNA oligonucleotides containing different unnatural bases, in which the original ssDNA oligonucleotide was removed by 5' phosphorylation and lambda exonuclease degradation. (**A**) Scheme for the sequencing of

ssDNA oligonucleotides containing different unnatural bases (**B**) Sanger sequencing results of the ssDNA oligonucleotides generated from ssDNA oligonucleotides containing a dNaM, dCNMO, or dTPT3 (T55-NaM, T55-CNMO, and T55-TPT3) and integral analysis for the percentages of the peak areas for different natural nucleotides at the positions of the unnatural bases. For each case, the sum of the peak areas of all four natural nucleotides was defined as 100%. In the sequence spectra for the selective conversion of T55-NaM with dATP (left) or dCTP (right). X: the position of dNaM in the original ssDNA oligonucleotide. In the sequence spectra for the selective conversion of T55-CNMO with dATP (left) or dCTP (right), X: the position of dCNMO in the original ssDNA oligonucleotide. In the sequence spectra for the selective conversion of T55-TPT3 with dTTP (left) or dCTP (right), X: the position of dTPT3 in the original ssDNA oligonucleotide.

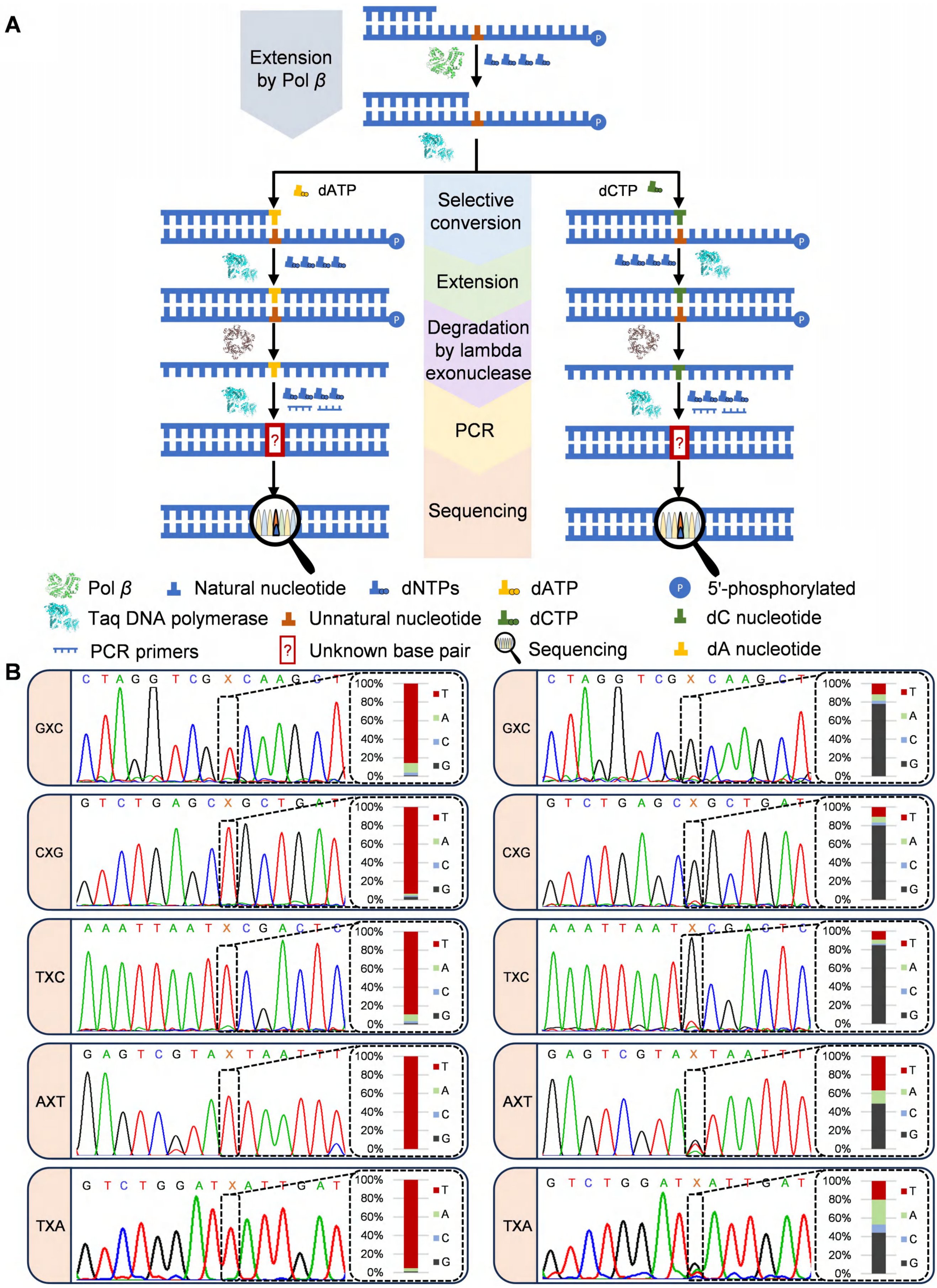

Figure S9. Pol β-mediated sequencing of ssDNA oligonucleotides containing a single

unnatural base and different upstream and downstream natural bases adjacent to the unnatural base, in which the original ssDNA oligonucleotide was removed by 5' phosphorylation and lambda exonuclease degradation. (A) Scheme for the sequencing of ssDNA oligonucleotides containing a single unnatural base and different upstream and downstream natural bases adjacent to the unnatural base. (B) Sanger sequencing results of the ssDNA oligonucleotides generated from ssDNA oligonucleotides containing a dNaM and different upstream and downstream natural bases adjacent to the dNaM [GXC: T40(GXC)-NaM; CXG: T71(CXG)-NaM; TXC: T70(TXC)-NaM; AXT: T70(AXT)-NaM; TXA: T71(TXA)-NaM] in which the selective conversion was carried out with dATP (left) or dCTP (right) and integral analysis for the percentages of the peak areas for different natural nucleotides at the positions of the unnatural bases. For each case, the sum of the peak areas of all four natural nucleotides was defined as 100%. X: the position of dNaM in the original ssDNA oligonucleotide.

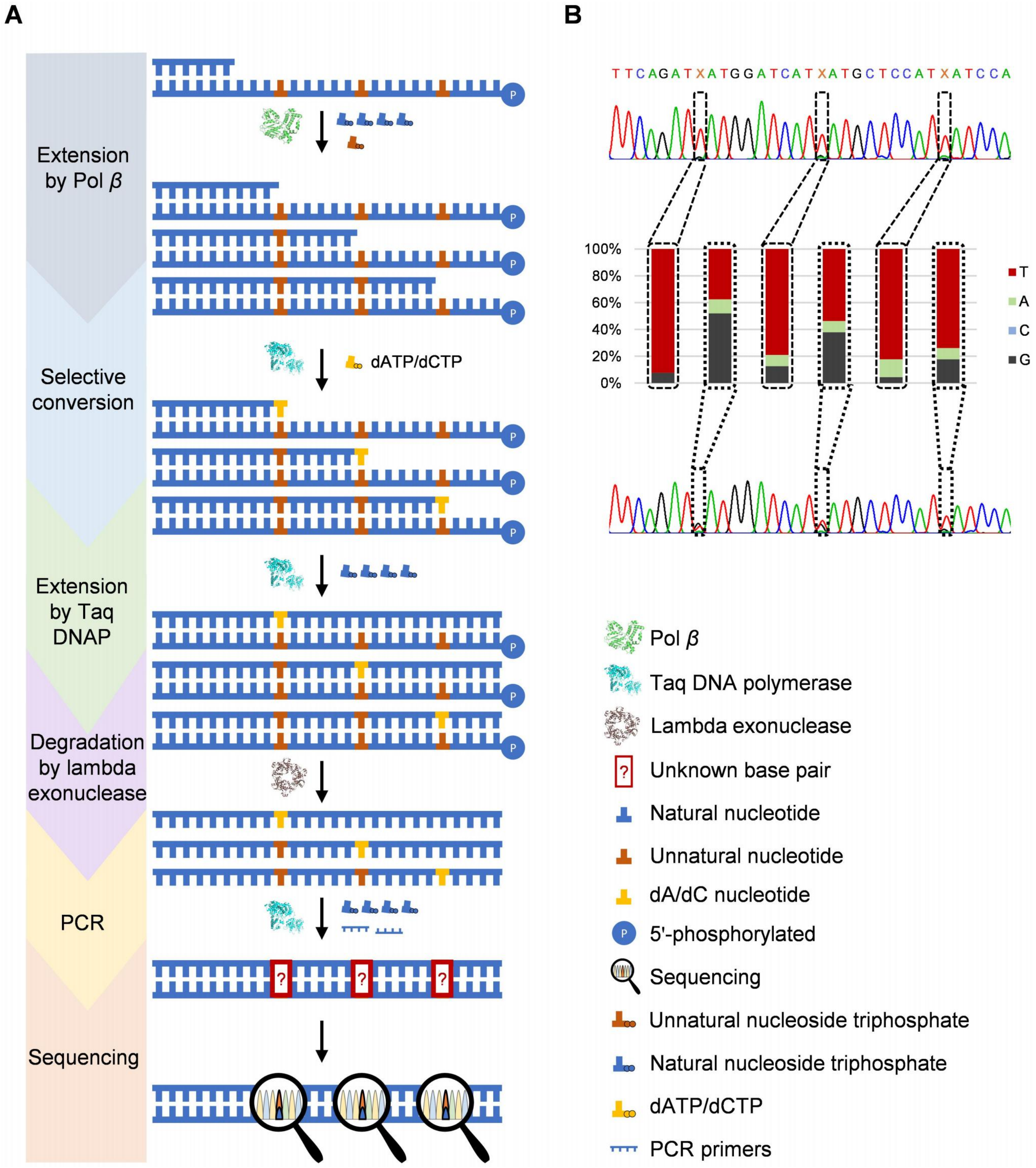

Figure S10. Pol *β*-mediated sequencing of an ssDNA oligonucleotide containing multiple unnatural bases, in which the original ssDNA oligonucleotide was removed by 5' phosphorylation and lambda exonuclease degradation. (**A**) Scheme for the sequencing of an ssDNA oligonucleotide containing multiple unnatural bases. (**B**) Sanger sequencing results for the sequencing of T79-NaM in which the selective conversion was carried out with dATP (upper) or dCTP (lower) and integral analysis for the percentages of the peak areas for

different natural nucleotides at the positions of the unnatural bases. For each case, the sum of the peak areas of all four natural nucleotides was defined as 100% X: the position of dNaM in the original ssDNA oligonucleotide.

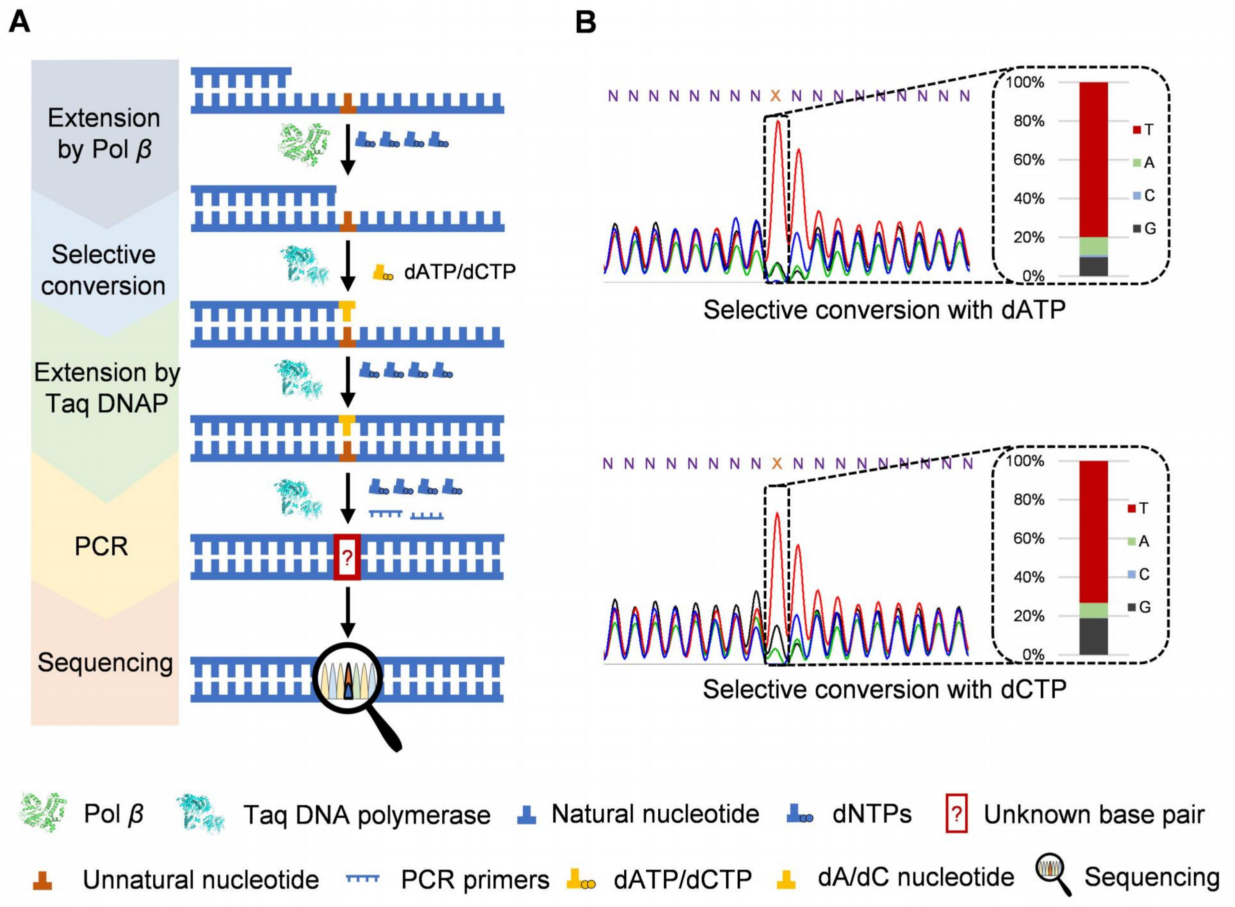

Figure S11. Pol *β*-mediated sequencing of a random ssDNA oligonucleotide library containing a single unnatural base, in which the original ssDNA oligonucleotide library was not removed by 5' phosphorylation and lambda exonuclease degradation. (**A**) Scheme for the sequencing of a random ssDNA oligonucleotide library containing a single unnatural base. (**B**) Sanger sequencing results when the selective conversion was carried out with dATP (upper) or dCTP (lower) and integral analysis for the percentages of the peak areas for different natural nucleotides at the positions of the unnatural bases. For each case, the sum of the peak areas of all four natural nucleotides was defined as 100%. X: the position of dNaM in the original ssDNA oligonucleotide.

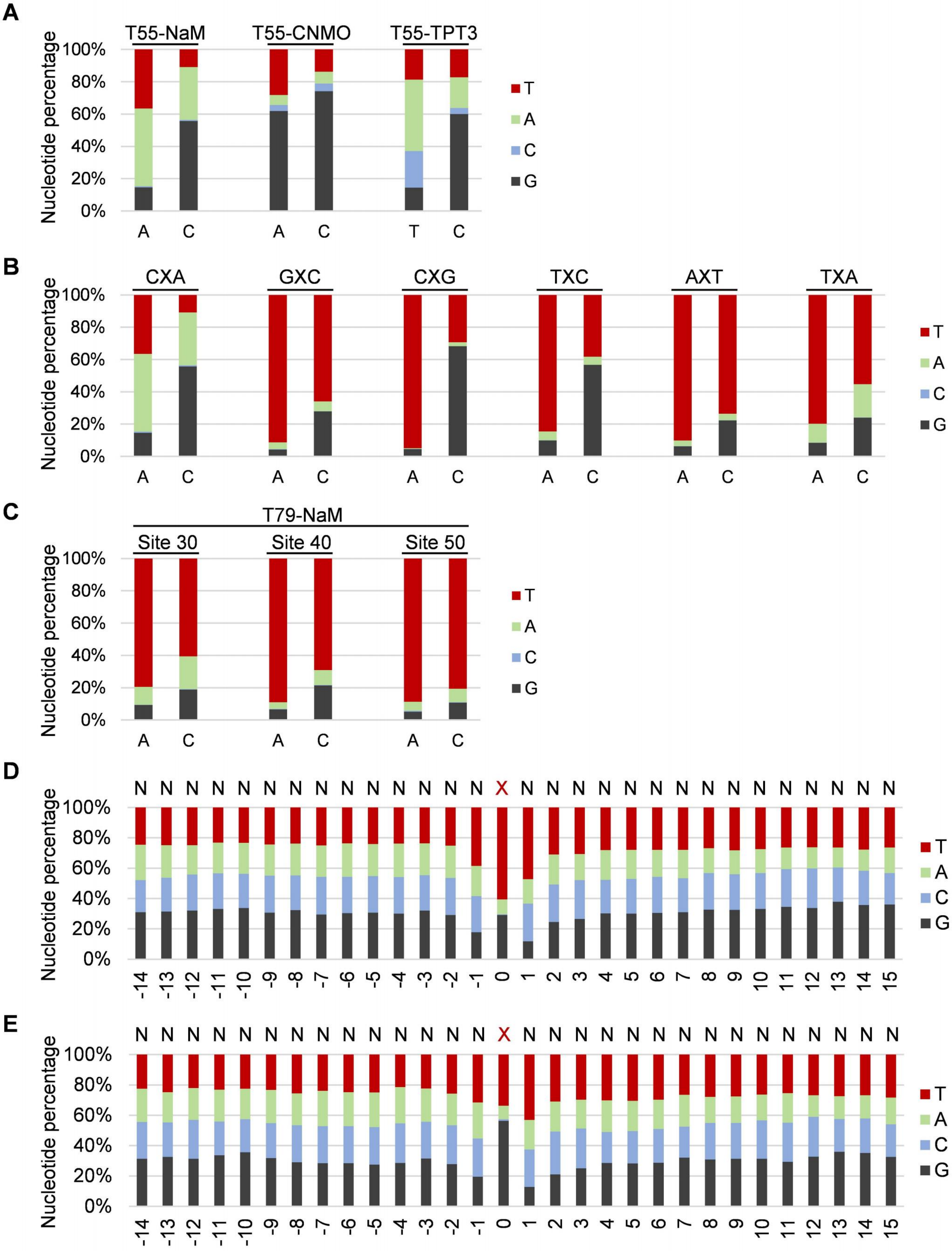

Figure S12. Analysis of the deep sequencing results for Pol β-mediated sequencing of ssDNA oligonucleotides or an ssDNA oligonucleotide library containing different unnatural bases, in

which the original ssDNA oligonucleotide or oligonucleotide library was not removed by 5' phosphorylation and lambda exonuclease degradation. (**A**) Percentages of different natural nucleotides at the position of the unnatural base from the deep sequencing results for Pol β-mediated sequencing of ssDNA oligonucleotides containing one unnatural base dNaM, dCNMO, or dTPT3 (T55-NaM, T55-CNMO, and T55-TPT3). A: selective conversion with dATP; C: selective conversion with dCTP; T: selective conversion with dTTP. (**B**) Percentages of different natural nucleotides at the position of the unnatural base from the deep sequencing results for Pol β-mediated sequencing of ssDNA oligonucleotides containing one unnatural base dNaM and different upstream and downstream natural bases adjacent to the dNaM. [CXA: T55-NaM; GXC: T40(GXC)-NaM; CXG: T71(CXG)-NaM; TXC: T70(TXC)-NaM; AXT: T70(AXT)-NaM; TXA: T71(TXA)-NaM]. A: selective conversion with dATP; C: selective conversion with dCTP. (**C**) Percentages of different natural nucleotides at the positions of the dNaMs (site 30, site 40, and site 50) from the deep sequencing results for Pol β-mediated sequencing of an ssDNA oligonucleotide containing multiple unnatural base dNaMs (T79-NaM). A: selective conversion with dATP; C: selective conversion with dCTP. (**D**) Percentages of different natural nucleotides at the positions of the unnatural base (0) and upstream and downstream natural bases (-14 to -1, 1 to 15) from the deep sequencing result for Pol β-mediated sequencing of a random ssDNA oligonucleotide library containing one unnatural base dNaM (LIB-NaM), in which the selective conversion was carried out with dATP. (**E**) Percentages of different natural nucleotides at the positions of the unnatural base (0) and upstream and downstream natural bases (-14 to -1, 1 to 15) from the deep sequencing result for Pol β-mediated sequencing of a random ssDNA oligonucleotide library containing one unnatural base dNaM (LIB-NaM), in which the selective conversion was carried out with dCTP. X: the position of dNaM in the original ssDNA oligonucleotide.

Supplementary Table

Table S1. Oligonucleotides used in this work.

Name	Sequence
FAM-P17	5'-FAM-CGTATGTTGTGTGGACT
Cy3-P17	5'-Cy3-CGTATGTTGTGTGGACT
T55-NaM	5'-CTGTTTCCTGTGTGAAATTGTTATGGTCTCTCNaMAGTCCACACAACATACGAAGCC
T55-TPT3	5'-CTGTTTCCTGTGTGAAATTGTTATGGTCTCTCTPT3AGTCCACACAACATACGAAGCC
T55-CNMO	5'-CTGTTTCCTGTGTGAAATTGTTATGGTCTCTCCNMOAGTCCACACAACATACGAAGCC
T55-A	5'-CTGTTTCCTGTGTGAAATTGTTATGGTCTCTCAAGTCCACACAACATACGAAGCC
T55-T	5'-CTGTTTCCTGTGTGAAATTGTTATGGTCTCTCTAGTCCACACAACATACGAAGCC
T55-C	5'-CTGTTTCCTGTGTGAAATTGTTATGGTCTCTCCAGTCCACACAACATACGAAGCC
T55-G	5'-CTGTTTCCTGTGTGAAATTGTTATGGTCTCTCGAGTCCACACAACATACGAAGCC
P20	5'-GAGAGACCATAACAATTTCA
5'-P-P20	5'-Pho-GAGAGACCATAACAATTTCA
P32	5'-GAATTCGAATTCTGAAATTGTTATGGTCTCTC
5'P-T43	5'-Pho-GGCTTCGTATGTTGTGTGGACTCGAGAGACCATAACAATTTCA
T40(GXC)-NaM	5'-ATCCGAATTCGAGCTAGGTCGNaMCAAGCTTGCGGCCGCACT
T71(CXG)-NaM	5'-ACTGGGACTGTGTGAAACCGACTTCAGTCTGGATNaMATTGATCGCTCCAGGACCGTCATAAAGTGTAAAGCC
T70(TXC)-NaM	5'-GCCCGAGATCTCGATCCCGCGAAATTAATNaMCGACT

	CACTATAGGGAGACCACAACGGTTTCCCTCTAGAA
T70(AXT)-NaM	5'-TTCTAGAGGGAAACCGTTGTGGTCTCCCTATAGTGAGTCGTANaMTAATTTCGCGGGATCGAGATCTCGGGCC
T71(TXA)-NaM	5'-ACTGGGACTGTGTGAAACCGACTTCAGTCTGGATNaMATTGATCGCTCCAGGACCGTCATAAAGTGTAAAGCC
T79-NaM	5'-ACTGGGACTGTGTGAAACCGACTTCAGATNaMATGGATCATNaMATGCTCCATNaMATCCAGGATCCGTCATAAAGTGTAAAGCC
LIB-NaM	5'-ACTGGGACTGTGTGAAACCGNNNNNNNNNNNNNNNaMNNNNNNNNNNNNNNNCCGTCATAAAGTGTAAAGCC
P20-30a	5'-TAGGAAGCAGCCCAGTAGTA
T47-1	5'-TCACACAGTCCCAGTGCGGCCGCAAATTTTAGCAGCAGCGGTTTCTT
FAM-P24-1	5'-FAM-GGCCTCGAGGGCTTTACACTTTAT
P27-1	5'-ATTTGCGGCCGCACTGGGACTGTGTGA
P47-2	5'-TCACACAGGAAACAGGCGGCCGCAAATTTTAGCAGCAGCGGTTTCTT
FAM-P24-2	5'-FAM-GGCCTCGAGGGCTTCGTATGTTGT
P27-2	5'-ATTTGCGGCCGCCTGTTTCCTGTGTGA
T47-3	5'-ATCGAGATCTCGGGCGCGGCCGCAAATTTTAGCAGCAGCGGTTTCTT
FAM-P24-3	5'-FAM-GGCCTCGAGTTCTAGAGGGAAACC
P27-3	5'-ATTTGCGGCCGCGCCCGAGATCTCGAT
P47-4	5'-GGTTTCCCTCTAGAAGCGGCCGCAAATTTTAGCAGCAGCGGTTTCTT
FAM-P24-4	5'-FAM-GGCCTCGAGGCCCGAGATCTCGAT
P27-4	5'-ATTTGCGGCCGCTTCTAGAGGGAAACC
T47-5	5'-AGCTCGAATTCGGATGCGGCCGCAAATTTTAGCAGCAGCGGTTTCTT

FAM-P24-5	5'-FAM-GGCCTCGAGAGTGCGGCCGCAAGC
P27-5	5'-ATTTGCGGCCGCATCCGAATTCGAGCT
Deep-T55NaM-A	5'-TAATACGACTCACTATAGGAAACTGTTTCCTGTGTGA
Deep-T55NaM-C	5'-TAATACGACTCACTATAGGAACCTGTTTCCTGTGTGA
Deep-T55CNMO-A	5'-TAATACGACTCACTATAGGACACTGTTTCCTGTGTGA
Deep-T55CNMO-C	5'-TAATACGACTCACTATAGGACCCTGTTTCCTGTGTGA
Deep-T55TPT3-T	5'-TAATACGACTCACTATAGGATACTGTTTCCTGTGTGA
Deep-T55TPT3-C	5'-TAATACGACTCACTATAGGATTCTGTTTCCTGTGTGA
Deep-T55-Down	5'-GGCTTTACACTTTATGACGGATCCGGCTTCGTATGTTGTG
Deep-P17	5'-GGCTTTACACTTTATGA
Deep-T79-A	5'-TAATACGACTCACTATAGGTTAACTGGGACTGTGTGA
Deep-T79-C	5'-TAATACGACTCACTATAGGTTCACTGGGACTGTGTGA
Deep-LIB-C	5'-TAATACGACTCACTATAGGTCCGGAATTGTGACTGGGACTGTGTGA
Deep-LIB-A	5'-TAATACGACTCACTATAGGTCAGGAATTGTGACTGGGACTGTGTGA
Deep-T40(GXC)-A	5'-TAATACGACTCACTATAGGAAAATTTGCGGCCGCATCCGAATTCGAGCTAGGTC
Deep-T40(GXC)-C	5'-TAATACGACTCACTATAGGAATATTTGCGGCCGCATCCGAATTCGAGCTAGGTC
Deep-T40(GXC)-Down	5'-GCCCCAAGGGGTTATGCTAGACAATTCCGGCCTCGAGAGTGCG
Deep-T71(CXG)-A	5'-TAATACGACTCACTATAGGNNNACTGGGACTGTGTGA
Deep-T71(CXG)-C	5'-TAATACGACTCACTATAGGTATACTGGGACTGTGTGA
Deep-T71(CXG)-Down	5'-GCCCCAAGGGGTTATGCTAGGGCTTTACACTTTAT
Deep-T70(TXC)-A	5'-TAATACGACTCACTATAGGGAAGCCCGAGATCTCGA

	T
Deep-T70(TXC)-C	5'-TAATACGACTCACTATAGGGATGCCCGAGATCTCGAT
Deep-T70(TXC)-Down	5'-GCCCCAAGGGGTTATGCTAGTTCTAGAGGGAAACC
Deep-T70(AXT)-A	5'-TAGGAAGCAGCCCAGTAGTACAATTCTAGAGGGAAACC
Deep-T70(AXT)-C	5'-TAGGAAGCAGCCCAGTAGTACATTTCTAGAGGGAAACC
Deep-T70(AXT)-Down	5'-GCCCCAAGGGGTTATGCTAGGCCCGAGATCTCGAT
Deep-T71(TXA)-C	5'-TAATACGACTCACTATAGGTACGGAATTGTACTGGGACTGTGTGA
Deep-T71(TXA)-A	5'-TAATACGACTCACTATAGGTAAGGAATTGTACTGGGACTGTGTGA

*FAM: FAM-labelled; Cy3: Cy3-labelled; Pho: phosphorylated.

Table S2. Percentages of the integrated peak areas for different natural nucleotides at the positions of the unnatural bases from the Sanger sequencing results for Pol *β*-mediated sequencing of the ssDNA oligonucleotides or oligonucleotide library containing one or multiple unnatural bases, in which the original ssDNA oligonucleotide or oligonucleotide library was not removed by 5' phosphorylation and lambda exonuclease degradation.

Sequenced oligonucleotide or oligonucleotide library	Position of the unnatural base in the oligonucleotide or oligonucleotide library	Nucleoside triphosphate used for selective conversion	Percentage of the integrated peak area for each natural nucleotide at the position of the unnatural base			
			T	A	C	G
T55-NaM	33	dATP	45.48%	41.38%	1.09%	12.06%
		dCTP	13.40%	31.37%	1.31%	53.92%
T55-CNMO	33	dATP	38.78%	10.53%	2.77%	47.92%
		dCTP	17.60%	14.31%	5.29%	62.80%
T55-TPT3	33	dTTP	21.76%	46.03%	20.71%	11.51%
		dCTP	21.23%	27.64%	8.29%	42.84%
T40(GXC)-NaM	22	dATP	83.86%	15.77%	0.00%	0.37%
		dCTP	46.13%	16.02%	5.27%	32.58%
T71(CXG)-NaM	35	dATP	95.69%	0.00%	1.97%	2.34%
		dCTP	32.99%	5.00%	2.73%	59.28%
T70(TXC)-NaM	30	dATP	64.20%	22.79%	2.21%	10.80%
		dCTP	27.23%	7.08%	1.33%	64.37%
T70(AXT)-NaM	43	dATP	86.41%	8.77%	3.24%	1.58%
		dCTP	51.82%	21.67%	2.04%	24.46%
T71(TXA)-NaM	35	dATP	76.42%	16.36%	0.00%	7.22%
		dCTP	48.53%	26.52%	1.20%	23.76%
T79-NaM	30	dATP	75.23%	13.54%	1.30%	9.93%

		dCTP	52.62%	20.13%	3.07%	24.19%
	40	dATP	81.91%	5.69%	0.09%	12.30%
		dCTP	55.64%	13.87%	3.01%	27.49%
	50	dATP	79.97%	6.76%	7.63%	5.64%
		dCTP	66.10%	12.27%	4.52%	17.10%
LIB-NaM	35	dATP	79.82%	9.12%	1.14%	9.92%
		dCTP	73.13%	7.71%	0.23%	18.93%

*For each case, the sum of the peak areas of all four natural nucleotides was defined as 100%.

Table S3. Percentages of the integrated peak areas for different natural nucleotides at the positions of the unnatural bases from the Sanger sequencing results for Pol *β*-mediated sequencing of the ssDNA oligonucleotides or oligonucleotide library containing one or multiple unnatural bases, in which the original ssDNA oligonucleotide or oligonucleotide library was removed by 5' phosphorylation and lambda exonuclease degradation.

Sequenced oligonucleotide or oligonucleotide library	Position of the unnatural base in the oligonucleotide or oligonucleotide library	Nucleoside triphosphate used for selective conversion	Percentage of the integrated peak area for each natural nucleotide at the position of the unnatural base			
			T	A	C	G
T55-NaM	33	dATP	91.67%	8.33%	0.00%	0.00%
		dCTP	17.53%	0.00%	0.00%	82.47%
T55-CNMO	33	dATP	66.80%	31.98%	0.00%	1.21%
		dCTP	0.00%	26.32%	0.00%	73.68%
T55-TPT3	33	dTTP	9.52%	90.48%	0.00%	0.00%
		dCTP	0.00%	30.43%	0.00%	69.57%
T40(GXC)-NaM	22	dATP	85.67%	10.27%	3.18%	0.87%
		dCTP	11.48%	7.01%	3.29%	78.21%
T71(CXG)-NaM	35	dATP	93.51%	1.95%	1.52%	3.03%
		dCTP	10.35%	6.23%	3.17%	80.25%
T70(TXC)-NaM	30	dATP	89.20%	7.48%	2.16%	1.16%
		dCTP	9.32%	4.41%	1.40%	84.87%
T70(AXT)-NaM	43	dATP	100.00%	0.00%	0.00%	0.00%
		dCTP	36.80%	14.38%	0.00%	48.82%
T71(TXA)-NaM	35	dATP	95.46%	3.43%	0.00%	1.17%
		dCTP	20.07%	26.99%	8.65%	44.29%
T79-NaM	30	dATP	92.42%	0.00%	0.00%	7.58%

		dCTP	37.50%	10.50%	0.00%	52.00%
	40	dATP	79.08%	8.31%	0.00%	12.62%
		dCTP	53.78%	8.37%	0.00%	37.85%
	50	dATP	82.21%	13.42%	0.00%	4.36%
		dCTP	73.86%	8.30%	0.00%	17.84%
LIB-NaM	35	dATP	66.60%	12.38%	1.88%	19.14%
		dCTP	25.20%	23.05%	1.50%	50.25%

*For each case, the sum of the peak areas of all four natural nucleotides was defined as 100%.

Table S4. Percentages of different natural nucleotides at the positions of the unnatural bases from the deep sequencing results for Pol *β*-mediated sequencing of the ssDNA oligonucleotides or oligonucleotide library containing one or multiple unnatural bases.

Sequenced oligonucleotide or oligonucleotide library	Position of the unnatural base in the oligonucleotide or oligonucleotide library	Nucleoside triphosphate used for selective conversion	Size of the sequencing library	Percentage of each natural nucleotide at the position of the unnatural base			
				T	A	C	G
T55-NaM	33	dATP	928769	36.60%	48.06%	0.79%	14.55%
		dCTP	922064	10.86%	32.78%	0.65%	55.71%
T55-CNMO	33	dATP	987155	28.15%	6.23%	3.76%	61.86%
		dCTP	921527	13.86%	7.27%	4.79%	74.08%
T55-TPT3	33	dTTP	1540617	18.68%	44.23%	22.66%	14.43%
		dCTP	1245881	17.19%	19.07%	3.72%	60.03%
T40(GXC)-NaM	22	dATP	447553	91.34%	4.23%	0.15%	4.28%
		dCTP	715909	65.93%	6.02%	0.15%	27.90%
T71(CXG)-NaM	35	dATP	782914	94.95%	0.34%	0.16%	4.55%
		dCTP	687265	29.38%	2.37%	0.09%	68.15%
T70(TXC)-NaM	30	dATP	708054	84.50%	5.49%	0.11%	9.90%
		dCTP	706574	38.21%	5.05%	0.04%	56.70%
T70(AXT)-NaM	43	dATP	989075	90.09%	3.46%	0.16%	6.28%
		dCTP	1004413	73.53%	4.11%	0.16%	22.20%
T71(TXA)-NaM	35	dATP	1447478	79.82%	11.65%	0.20%	8.33%
		dCTP	1271832	55.34%	20.62%	0.14%	23.90%
T79-NaM	30	dATP	239641	79.50%	11.02%	0.16%	9.32%
		dCTP	265877	60.65%	20.30%	0.27%	18.78%
	40	dATP	239641	88.95%	4.24%	0.28%	6.53%

		dCTP	265877	69.05%	9.22%	0.35%	21.38%
	50	dATP	239641	88.67%	5.60%	0.60%	5.13%
		dCTP	265877	80.60%	8.35%	0.35%	10.70%
LIB-NaM ($lambda^+$)	35	dATP	6625	60.65%	9.75%	0.50%	29.10%
		dCTP	1945	33.68%	8.95%	0.98%	56.40%

Supplementary Note

Note S1. Protein sequence of Pol *β*.

MSKRKAPQETLNGGITDMLTELANFEKNVSQAIHKYNAYRKAASVIAKYPHKIKSGA

EAKKLPGVGTKIAEKIDEFLATGKLRKLEKIRQDDTSSSINFLTRVSGIGPSAARKFVD

EGIKTLEDLRKNEDKLNHHQRIGLKYFGDFEKRIPREEMLQMQDIVLNEVKKVDSEY

IATVCGSFRRGAESSGDMDVLLTHPSFTSESTKQPKLLHQVVEQLQKVHFITDTLSKG

ETKFMGVCQLPSKNDEKEYPHRRIDIRLIPKDQYYCGVLYFTGSDIFNKNMRAHALE

KGFTINEYTIRPLGVTGVAGEPLPVDSEKDIFDYIQWKYREPKDRSE
